# Pandemic personality: Emotional reactions, political and social preferences across personality traits in times of Corona

**DOI:** 10.1007/s12144-021-02493-x

**Published:** 2021-11-23

**Authors:** Markus Freitag, Nathalie Hofstetter

**Affiliations:** Institute of Political Science, University of Bern, Fabrikstrasse 8, CH-3012 Bern, Switzerland

**Keywords:** Personality, Pandemic, Big five, Comparative politics, Emotions

## Abstract

The Coronavirus pandemic undeniably represents a global health threat unprecedented in living memory leading to very distinct behavioral, cognitive, and psychological responses to the crisis. We argue that the different ways of responding to the pandemic are rooted in personal dispositions and provide evidence regarding the function and value of the Big Five framework in understanding the pandemic personality. Using 18 samples from the six European countries most affected at the onset of the pandemic (overall N = 18,307), we find that most of the Big Five effects vary across countries and pandemic phases. However, while neuroticism is clearly linked to pandemic threat perception and emotional responses to the Covid-19 pandemic, conscientiousness is mainly related to exposure to pandemic hazard, preferences regarding political measures, and tolerance of epidemiologically undesirable behavior. Our findings are rich in implications for public health politics, policy-makers and social cohesion.

**Supplementary Information:**

The online version contains supplementary material available at 10.1007/s12144-021-02493-x.

## Introduction

According to Johns-Hopkins-University, by November 2021 more than 250 million people have been infected with Coronavirus (Johns Hopkins University, 2021). Covid-19 is threatening because it seems uncontrollable and evokes a citizen’s fears of a novel and deadly illness that could spread rapidly among many people (Kachanoff et al., 2020). This sense of threat comes with a high degree of uncertainty, relating both to the nature of the pandemic and to its consequences: Uncertainty about getting infected, uncertainty about whether the people around you are infected, uncertainty about the ideal form of protective measures, uncertainty about new virus mutations, and uncertainty about whether the pandemic is truly eliminated (Taylor, 2019, p. 43). If “individual differences tend to be accentuated in settings characterized by novelty, ambiguity, and uncertainty” (Caspi & Moffitt, 1993, p. 266), then individuals should differ particularly in their responses to the Covid-19 pandemic.

Hitherto, several studies point to a link between individual differences and Covid-19 related criteria, regarding for example compliance with Covid-19 containment measures, the development of depressive symptoms, or toilet paper stockpiling (Aschwanden et al., 2021; Asselmann et al., 2020; Blagov, 2021; de F. Carvalho et al., 2020; Chan et al., 2021; Garbe et al., 2020; Götz et al., 2020; Han, 2021; Kroencke et al., 2020; Nikčević et al., 2021; Qian & Yahara, 2020; Zajenkowski et al., 2020). We enrich this relevant literature in three important ways.

- First, compared to previous studies, our research interest lies primarily with emotional responses and social and political preferences. So far, little is known about how personality shapes the evaluation of Covid-19 measures or attitudes toward people who do not comply with them. However, these aspects have seminal implications for policy makers as well as for social integration and cohesion. Moreover, to the best of our knowledge, there is scant empirical evidence on whether and how personality is related to exposure to the viral hazard. However, such information can help to identify people who are particularly at risk of infection and target them with specific measures and appeals that resonate with their personalities.
- Second, previous findings of the above mentioned studies are characterized by a substantial lack of external validity, as most studies are single-country analyses or use student and other convenience samples. What is more, the few exceptions carrying out cross-country analyses (e.g. Chan et al., 2021; Götz et al., 2020; Zettler et al., 2021) often forego country-specific analyses by simply pooling data from different contexts which can obscure important differences between them (cf. Weinschenk, 2017). In the present study, we not only significantly expand the empirical basis for examining personality effects on Covid-19-relevant outcomes by using 18 samples from six European countries (overall N = 18,307), we provide a more fine-grained picture of these effects by examining them separately across different country contexts. Taking into account the situation at the onset of the crisis in early spring 2020, our surveys include respondents from Italy, France, Germany, Spain, the United Kingdom and Switzerland. These six countries were the most severely hit in Europe until mid-April 2020 (for Covid-19 relevant data see WHO, 2020a, 2020b). By looking at these countries comparatively, we contribute to the important question of how generalizable or context-dependent personality effects are and find both cross-nationally robust and variable personality effects on Covid-19-relevant outcomes.
- Third, we address the hitherto understudied effect of personality effects over time (Weinschenk, 2017, p. 1418) by considering three time points during the pandemic in Europe: the initial Corona wave in spring 2020, the second pandemic escalation in winter 2020/2021, and the broad vaccination phase in spring 2021. Consistent with cautious comments about drawing conclusions based on current Covid-19 social science research, as well as lessons from the “replicability crisis” in psychology and beyond, identifying robust, replicable, and generalizable evidence seems critical (Zettler et al., 2021).

In our data, we identify the pandemic personality by arriving at the following main findings: First, neuroticism, more than any other trait, robustly predicts mental states during the pandemic. We find this trait relates to heightened perceptions of health, financial and social threat as well as to higher levels of fear and anger throughout the Covid-19 crisis. Second, there is robust evidence that the personality dimension of conscientiousness is related to greater protection against the virus by lowering infection risk, tolerance for epidemiologically undesirable behavior and by pushing for collective security when it comes to governmental containment measures. Third, the remaining three personality dimensions exert less clear and robust effects. Fourth, most of the Big Five effects vary across countries and pandemic phases, underlining the relevance of explicitly considering the context-dependency or conditionality of personality effects.

## Emotional Reactions, Pandemic Exposure, and Political and Social Preferences across Personality Traits

According to the trait paradigm, an individual’s personality can be described as the entirety of all characteristics reflecting “relatively stable patterns of feeling, thinking, striving, and behaving and by which a person is more or less distinguished from others [...]” (Kandler & Riemann, 2015, p. 51). Personality traits are understood as the core components of a relatively enduring and multifaceted internal personality system, shaping how individuals respond to the vast array of stimuli they encounter in the world (Gerber et al., 2011; Mondak, 2010, p. 86). Traits cannot be observed directly but are inferred from behavior; they are found to be considerably stable over the course of life and situations, and are at least partly determined by genetic dispositions (McCrae & Costa, 2008, p. 162; Mondak, 2010, p. 7). In order to comprehensively conceptualize and reliably measure personality traits, the Big Five or Five Factor Model has emerged as the dominant framework in psychology in recent years (Freitag & Rapp, 2015; Gerber et al., 2011; Mondak & Halperin, 2008). As a “general taxonomy of personality traits” (John et al., 2008, p. 116), the model comprises five superior and abstract personality dimensions, the so-called Big Five – openness to experience, conscientiousness, extraversion, agreeableness and neuroticism – that emerge across different cultural and linguistic contexts (Gallego & Oberski, 2012; Gallego & Pardos-Prado, 2014; McCrae & Costa, 2008). Openness to experience refers to a curiosity about new experiences, ideas and actions and is usually conveyed by adjectives such as open-minded, interested, nonconforming and tolerant (Caprara & Vecchione, 2013; Mondak & Halperin, 2008). Conscientious individuals are organized, responsible, reliable, dutiful and highly appreciate control, structure and conformity (Gallego & Pardos-Prado, 2014; Mondak & Halperin, 2008; Weinschenk, 2017). Extraversion describes an energetic and excitement-seeking approach toward life and includes sociability, positivity and activity (Dinesen et al., 2016; Gallego & Pardos-Prado, 2014; Gerber et al., 2011). Agreeableness refers to a prosocial and communal orientation to others, to conflict-aversion and a concern for the well-being of society as a whole. People scoring high on this trait are typically described as caring, cooperative, compliant, tolerant and trusting (Gallego & Pardos-Prado, 2014; Gerber et al., 2011; Roccas et al., 2002). Finally, neuroticism contrasts emotional stability and refers to the control of impulses and emotions, commonly conveyed by adjectives such as anxious, tense, worried, and vulnerable (Caprara & Vecchione, 2013; Fatke, 2017; Mondak & Halperin, 2008). As these five personality traits relate to attitudinal and behavioral tendencies in all spheres of life (McCrae & Costa, 2008), we also expect them to influence pandemic exposure and shape the way individuals emotionally and cognitively respond to the current Covid-19 pandemic. In many cases, there are specific expectations about the likely relationships between the Big Five factors and pandemic threat perception, emotional responses, exposure to pandemic hazard, preferences regarding political measures, and tolerance of epidemiologically undesirable behavior. In other cases, where previous research provides only rough indications, our expectations are less concrete and more exploratory in nature.

To begin with, we can expect *open* individuals to be curious, interested and informed in what is going on, and generally coping fairly well with the adjustment to the new situation (Aschwanden et al., 2021, p. 52; Asselmann et al., 2020). In other words: We may assume that acquired knowledge about the pandemic limits the feelings of uncertainty and insecurity of open-minded people and therefore reduces their feelings of threat and anxiety. Moreover, since open individuals are critical citizens, tend to reject state intervention and hold (socially) liberal values, they should be more prone to reject far-reaching and restrictive political measures (Cooper et al., 2013; Gerber et al., 2011). Open-mindedness further implies a general receptiveness to new opinions, values, beliefs as well as alternative lifestyles and choices (Christensen, 2020, p. 4; Cooper et al., 2013, p. 71; Freitag & Rapp, 2015). With this in mind, we also expect open-minded people to be tolerant of people who disregard Covid-19 measures.

As *conscientious* individuals are typically very disciplined, rule-consistent, responsible and cautious, one could expect them to rigidly support far-reaching policy measures and governmental rules to fight the spread of the virus and change their behavior accordingly (Asselmann et al., 2020, p. 2; Blagov, 2021; Brouard et al., 2020; de F. Carvalho et al., 2020; Han, 2021). Correspondingly, the rule-abiding behavior of conscientious individuals should be associated with a lower risk of exposure or infection. However, the highly extraordinary and somewhat confusing situation of the Coronavirus crisis is likely to evoke negative emotions and be perceived as threatening by conscientious individuals who need structure and seek to retain control over any given situation. Moreover, due to their pronounced demand for conformity (Kunst et al., 2021; Mondak & Halperin, 2008), conscientious people should not tolerate non-compliance with the enacted social rules and norms to prevent the spread of the disease.

Favoring hierarchical structures and strong political leadership, *extraverts* are expected to support far-reaching containment policies. However, because of their outgoing and sociable nature, extraverted individuals should have particular difficulties eliminating social contacts and activities during the Covid-19 pandemic, and should thus engage less strictly in social distancing measures or ‘stay-home’ recommendations (Asselmann et al., 2020, p. 2). This is strongly supported by current findings which indicate that extroverts do not comply with containment measures (e.g. Brouard et al., 2020; Chan et al., 2021; Götz et al., 2020; Han, 2021). Therefore, it is likely that extraverted people are particularly exposed to the pandemic as they do not protect themselves properly and will also tend to meet more infected people due to their large social networks. In addition, these individuals are less critical and instead rather tolerant of others who exhibit deviant behavior. Considering the positivity of extraverts, it is rather unlikely that they are plagued by great fear, anger, or other negative feelings in light of the pandemic (cf. Agbaria & Mokh, 2021; Nikčević et al., 2021).

Given their inclination for cooperation, solidarity, and concern for the well-being of society as a whole (Gerber et al., 2011, p. 267), we expect *agreeable* people to comply more strictly with rules and recommendations, which could make them less prone to infection (Asselmann et al., 2020, p. 2; Blagov, 2021; Chan et al., 2021; Götz et al., 2020; Han, 2021). In general, agreeable people should prefer collective security to individual freedoms. Thus, measures to contain the pandemic could hardly go far enough for these cooperative and caring individuals. Although there is ample empirical evidence regarding the generally tolerant, permissive and understanding nature of agreeable people (Freitag & Rapp, 2015; Gallego & Pardos-Prado, 2014), we might suspect that agreeable individuals will reject behavior that runs counter to the collective goals and efforts, and be correspondingly intolerant of those who do not comply with the prescribed measures. They should also perceive the pandemic as a threat to community life and public health, inducing fear. Other negative emotions, such as anger, however, do not seem to fit the conciliatory and harmonious nature of these individuals.

Due to their tendency toward hyper-concern and emotional vulnerability, *neurotics* are expected to react in a markedly negative way emotionally to the current pandemic (cf. Agbaria & Mokh, 2021; Kroencke et al., 2020; Nikčević et al., 2021). We could therefore further assume that these people feel particularly threatened in the context of Covid-19 (cf. Aschwanden et al., 2021; Asselmann et al., 2020; Garbe et al., 2020). Moreover, recent studies show that the generally risk-averse neurotics protect themselves from infection in many ways, sometimes even going beyond governmental measures and recommendations (e.g. Asselmann et al., 2020; Blagov, 2021; Chan et al., 2021; Garbe et al., 2020; Götz et al., 2020; Qian & Yahara, 2020).^1^ Because of their high need for security, neurotic individuals should be more likely to support policies that limit the risk of infection. The social isolation accompanying this behavior should reduce their exposure to the pandemic threat accordingly. Finally, due to their own integrity and fears, individuals scoring high on neuroticism should prove to be strict and uncompromising, thus being less tolerant towards those exhibiting socially undesirable behavior.

## Data and Methods

To test the various expectations outlined above, we rely on original cross-sectional survey data of over 18,000 European respondents collected at three time points during the Coronavirus pandemic in Europe. Taking into account the epidemiological situation at the onset of the pandemic, each of our 18 samples contains about 1000 individuals from Italy, France, Germany, Spain, the United Kingdom and Switzerland as these countries were the most severely hit in Europe at the time (WHO, 2020a, 2020b). The first approximately 6000 individuals were surveyed during the initial peak of the pandemic in spring 2020, between April 17 and May 11. We collected data on another 6000 Europeans during the second pandemic escalation in winter 2020/2021, from November 24 to January 18, and finally again during the broad vaccination phase in spring 2021, between April 22 and May 21.^2^ The first large online survey was conducted by Qualtrics, the latter two by SurveyEngine. Quota on age, gender and education for each country (including language for Switzerland) were used for all surveys to mirror the distribution of these variables representative for the entire population (see Table AT0 in the online appendix for a description of the surveys).

While we will discuss the measurement of our broad set of dependent variables (e.g., pandemic threat perception, emotional responses, exposure to pandemic hazard, political preferences regarding political measures, and tolerance of epidemiologically undesirable behavior) in each of the corresponding sections, the Big Five personality traits are considered as explanatory variables in all subsequent analyses. We use Gosling et al. (2003)‘s highly influential Ten-Item Personality Inventory (TIPI) to create arithmetic means from the related items. Albeit a considerably short scale, psychometric evidence – e.g. regarding test-retest reliability, convergent validity, or factor structure – suggests that the TIPI is an appropriate measure of the Big Five (Nunes et al., 2018, p. 2; Romero et al., 2012, p. 289). Like the original version of Gosling et al. (2003), we find the strongest internal consistency estimates for conscientiousness, extraversion, and neuroticism. For openness to experience and agreeableness, Cronbach’s alpha and Spearman-Brown estimates are lower.^3^ Following Mondak (2010, p. 72), the indices were logarithmized to minimize the impact of social desirability effects (for the distribution of the personality traits among pandemic phases and the six countries see Tables AT1 & AT2 in the online appendix). As controls we include gender, age, education and income situation in all our analyses (Stapleton et al., 2021) (for descriptive statistics see Table AT3 in the online appendix).

## Empirical Analysis

### Question 1: Who Feels Particularly Threatened by the Covid-19 Pandemic?

To assess the perceived threat from Covid-19, we use three different items referring to potentially threatening aspects of the pandemic. Asking the respondents how worried they are that they, a family member or someone from their immediate circle could become infected with Coronavirus indicates their perceived level of *health threat*. Furthermore, we asked respondents how they perceive the pandemic as a threat to their own financial situation (*financial threat*) as well as to their social relationships (*social threat*). Answer scales range from 1 “not very worried” to 4 “very worried” (figures AF1-AF3 in the online appendix graphically depict the levels of perceived threats across countries and time points).

**Fig. 1:**
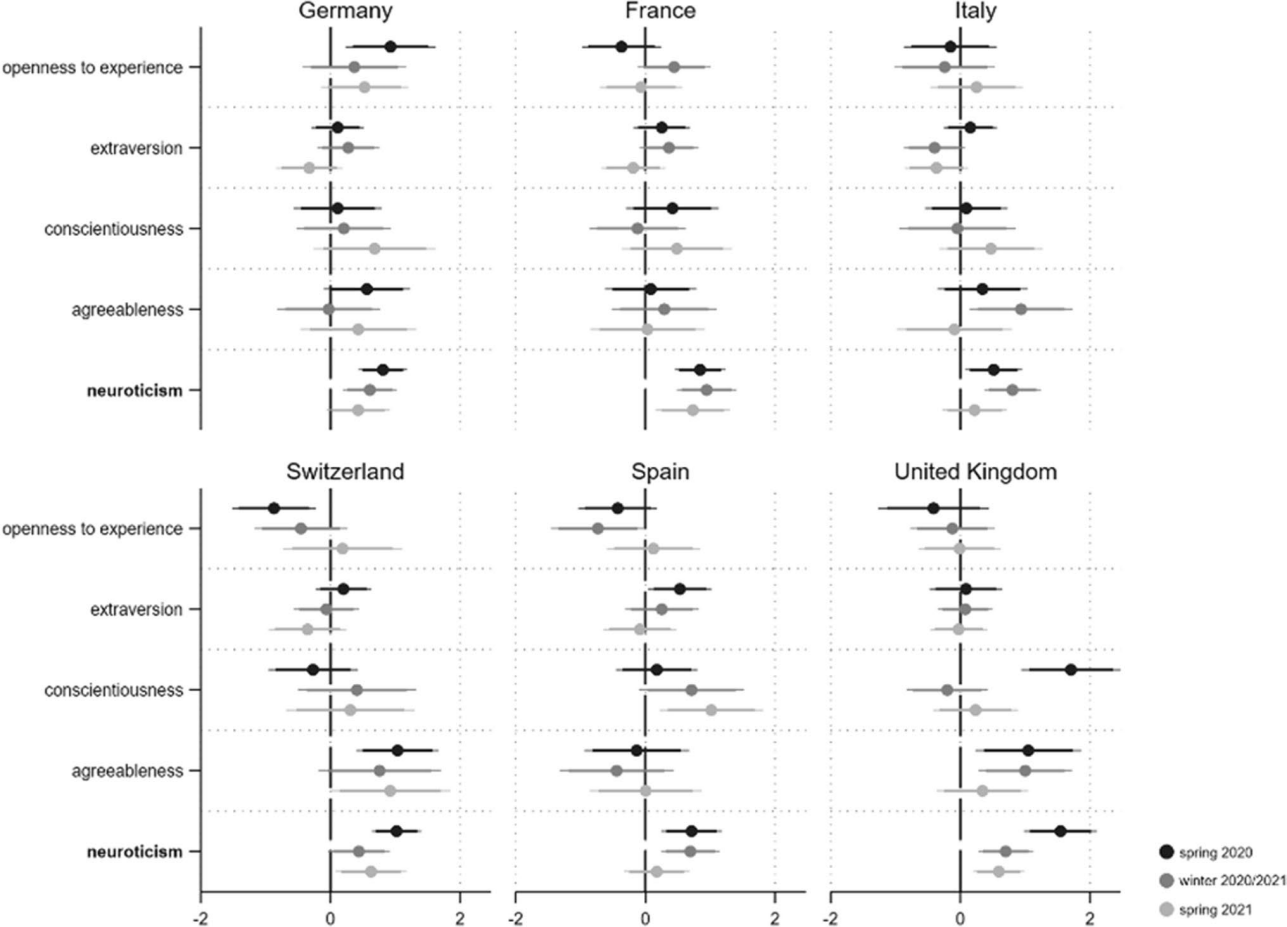
Personality and perceived health threat from Covid-19. *Note*: Displayed are the ordered logit regression coefficients (markers) with their 95 and 90% confidence intervals (horizontal lines). Personality traits with relatively robust effects are in bold. Models (fully presented in AT4a/b) control for gender, age, education and income situation

**Fig. 2:**
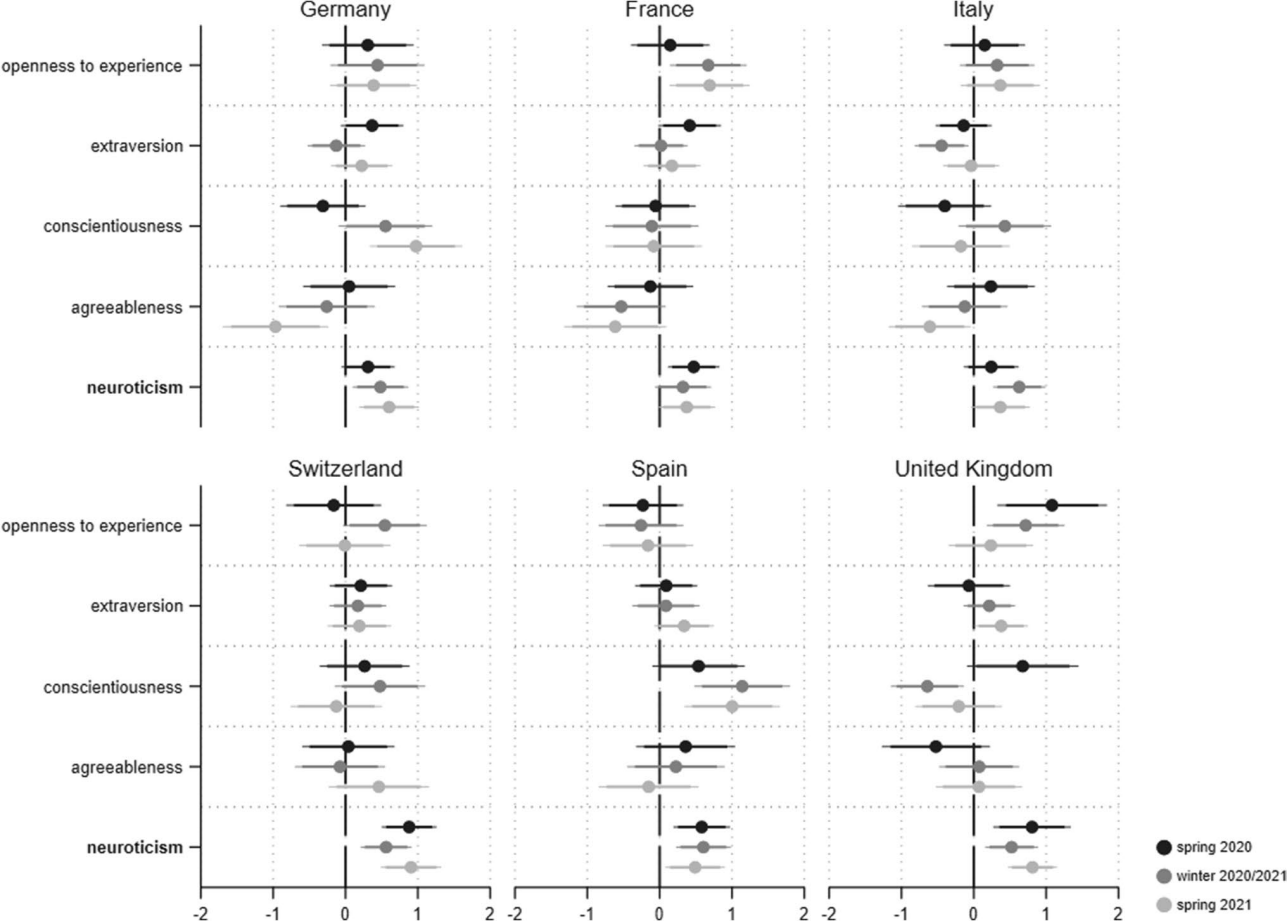
Personality and perceived financial threat from Covid-19. *Note*: Displayed are the ordered logit regression coefficients (markers) with their 95 and 90% confidence intervals (horizontal lines). Personality traits with relatively robust effects are in bold. Models (fully presented in AT5a/b) control for gender, age, education and income situation

**Fig. 3:**
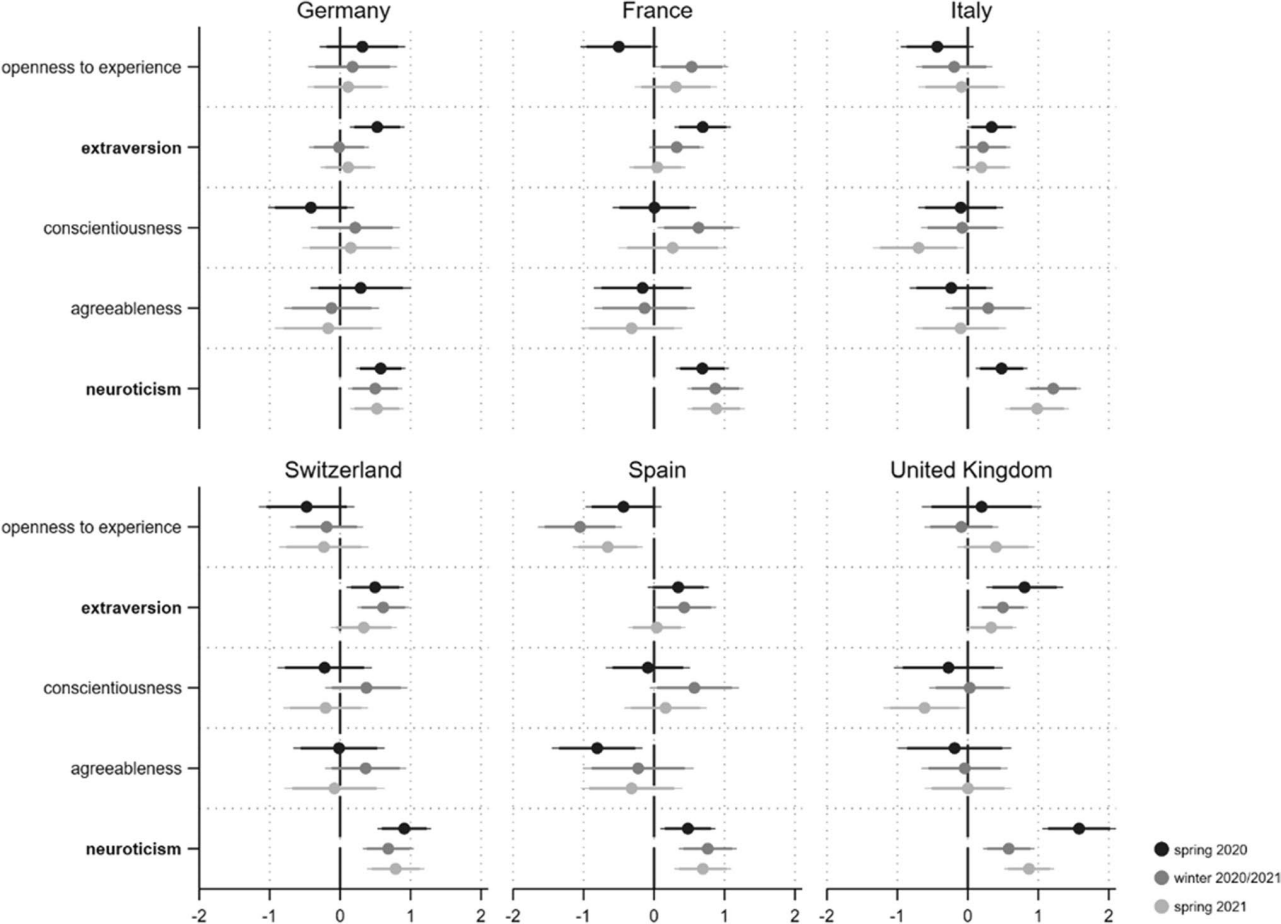
Personality and perceived social threat from Covid-19. *Note*: Displayed are the ordered logit regression coefficients (markers) with their 95 and 90% confidence intervals (horizontal lines). Personality traits with relatively robust effects are in bold. Models (fully presented in AT6a/b) control for gender, age, education and income situation

Conducting ordered logit regressions, we find evidence that neuroticism in particular is consistently related to the different threat aspects (see Figs. 1, 2, 3).^4^ First, the more neurotic a person, the more they perceive the pandemic as threatening regarding its health consequences. Across the 18 coefficients for this relationship, 16 are statistically significant (14 coefficients p < 0.05 and two p < 0.1). For agreeableness, one third of the respective coefficients reach statistical significance (four coefficients p < 0.05 and two p < 0.1). Other personality traits do not substantially relate to perceived health threat during the pandemic.

Regarding the financial consequences of the pandemic (see Fig. 2), neuroticism again leads to heightened concern in almost every country and at every time point. Besides the unambiguous effect of neuroticism, we find weak evidence that people scoring high on conscientiousness also feel more financially threatened.

This effect is particularly evident in Spain over the course of the pandemic, whereas we find no such an effect in France, Italy, or Switzerland.^5^ Finally, the crucial role of neuroticism is most impressively demonstrated with respect to social threat (see Fig. 3): Here, all 18 coefficients are positive and statistically significant (p < 0.05). The current crisis is also perceived as a greater social threat by extraverts which is not surprising given their sociable nature (six coefficients p < 0.05 and three p < 0.1). This relationship is most explicit in the United Kingdom where the closure and reopening of pubs as central social anchors was highly disputed. However, the effect is much less robust among countries and pandemic phases.

### Question 2: Who Feels Anger and Fear in Response to Covid-19?

**Fig. 4:**
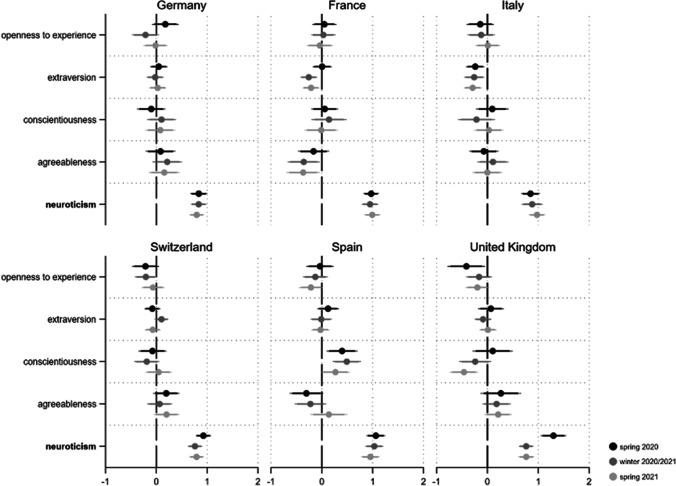
Personality and fear during the Covid-19 pandemic*. Note*: Displayed are linear regression coefficients (markers) with their 95 and 90% confidence intervals (horizontal lines). Personality traits with relatively robust effects are in bold. Models (fully presented in AT7a/b) control for gender, age, education and income situation

**Fig. 5:**
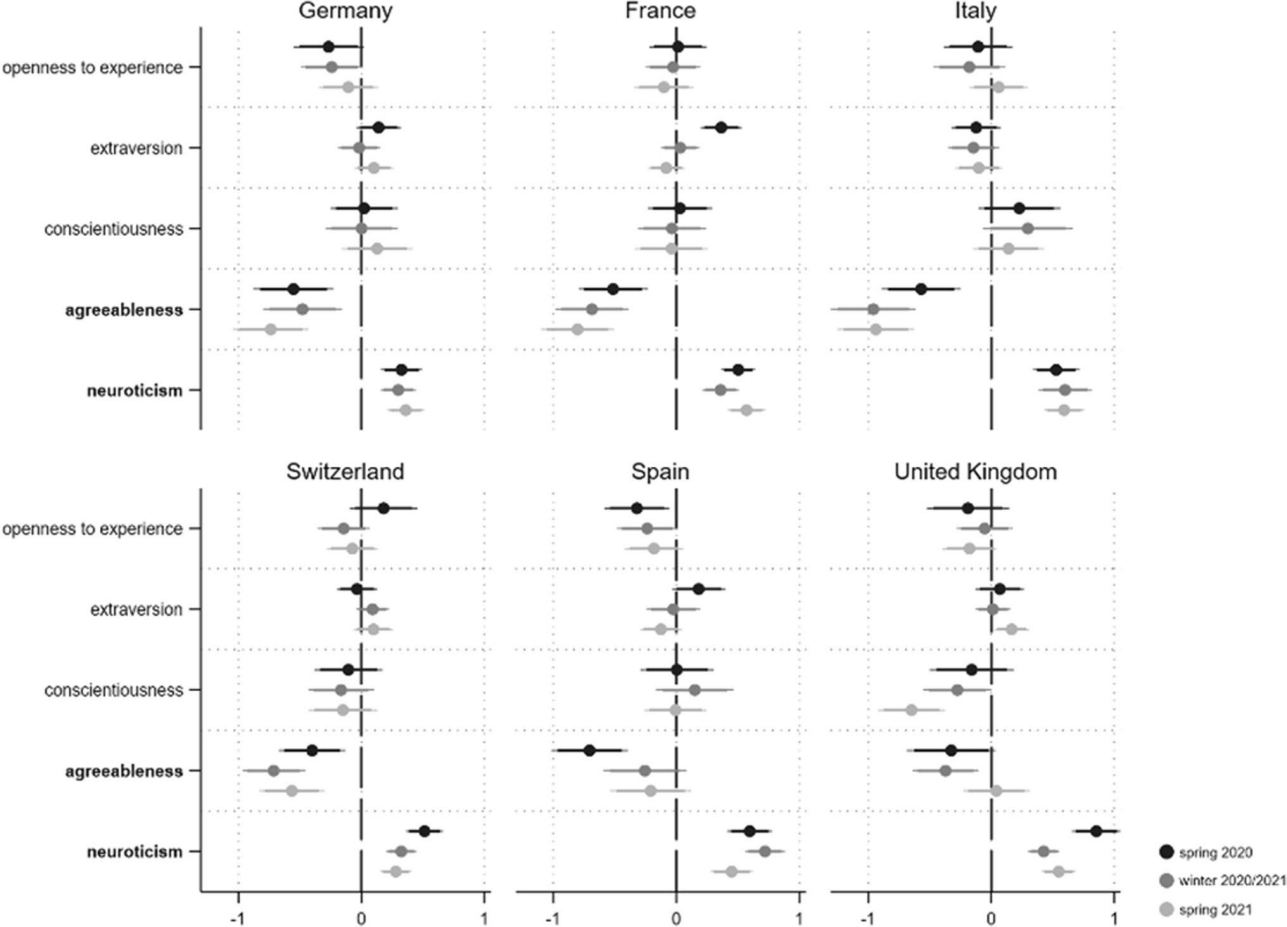
Personality and anger during the Covid-19 pandemic*. Note*: Displayed are linear regression coefficients (markers) with their 95 and 90% confidence intervals (horizontal lines). Personality traits with relatively robust effects are in bold. Models (fully presented in AT8a/b) control for gender, age, education and income situation

According to the relevant literature, the emotional experiences of fear and anger are the typical affective reactions to a threatening situation (Brader & Marcus, 2013; Marcus et al., 2000, 2019).^6^ To measure these main dimensions of emotions, we rely on the well-known Positive and Negative Affect Schedule (PANAS) scale in its short version (Crawford & Henry, 2004; Watson et al., 1988). Respondents were asked to indicate, on a scale from 1 (‘not at all’) to 5 (‘extremely’), how intensely they experience a list of different emotions and feelings at the moment. For fear we included the emotive terms ‘afraid’ and ‘nervous’, while for anger we used the emotional states of being ‘hostile’ and ‘upset’.^7^ Figures AF4 and AF5 in the online appendix show the average levels of experienced fear and anger across countries and time points. As for perceived threats from Covid-19, we again find the personality dimension of neuroticism to be of particular relevance for emotional reactions to the crisis. Regarding fear and anger, we find this trait to be the only one consistently related to these negative emotional experiences (see Figs. 4 and 5): The more neurotic a person, the more they experience fear and anger in times of the pandemic.

All 36 respective coefficients are highly significant, implying that the relationships are highly robust across the countries and pandemic phases under study. With regard to anger, we additionally find agreeableness to play a substantial role (14 coefficients p < 0.05 and one p < 0.1). Being more critical, quarrelsome and intolerant thus goes hand in hand with increased anger during the pandemic, particularly in Germany, France, Italy and Switzerland.^8^

### Question 3: Who is at Particular Risk of a Covid-19 Infection?

**Fig. 6:**
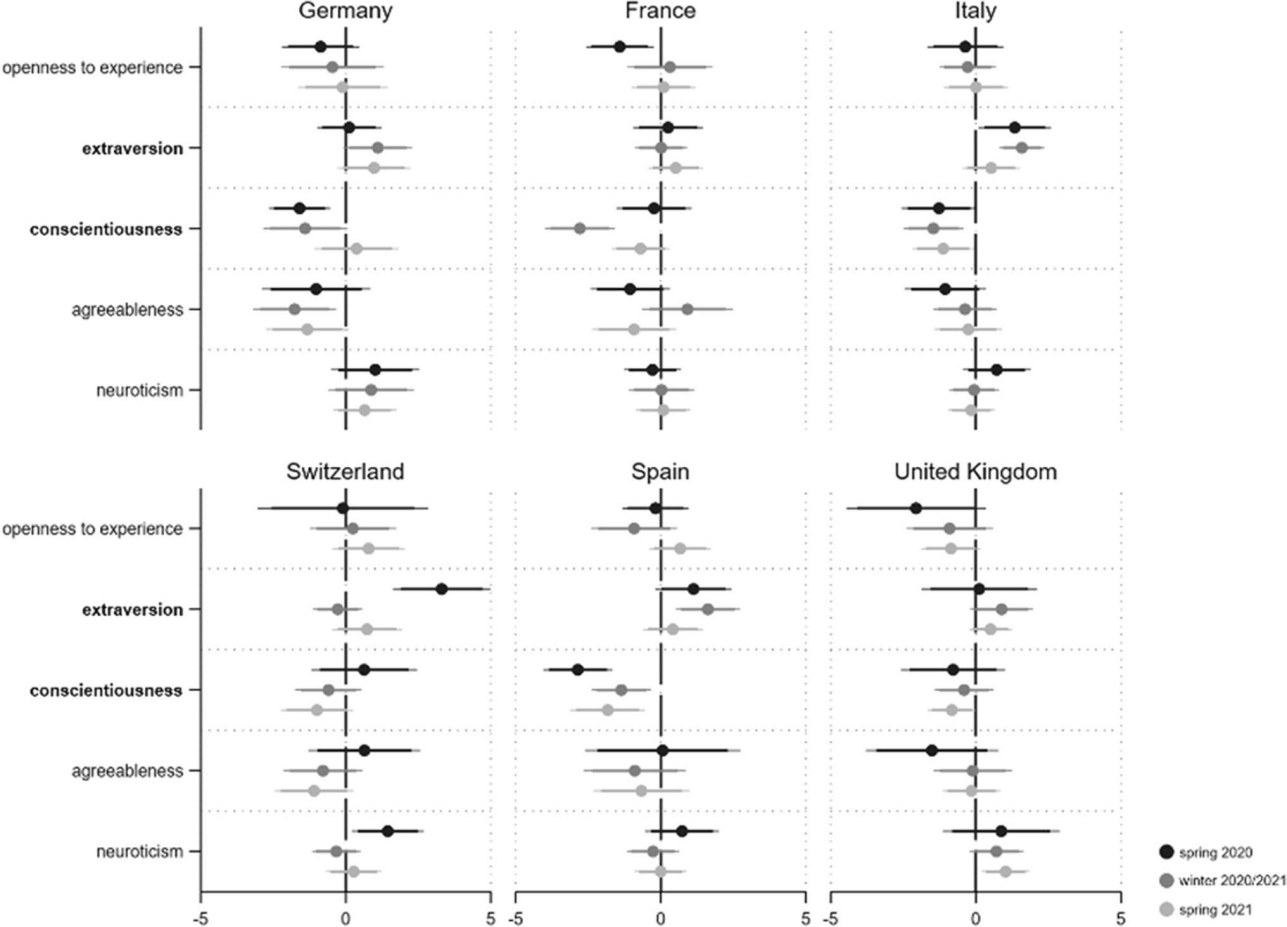
Personality and Covid-19 self-infection*. Note*: Displayed are logistic regression coefficients (markers) with their 95 and 90% confidence intervals (horizontal lines). Personality traits with relatively robust effects are in bold. Models (fully presented in AT9a/b) control for gender, age, education and income situation

As a next step we are interested in whether our personality drives exposure to the pandemic. As an indicator of exposure, we use *self-infection*. Respondents indicating that they have already been diagnosed with Coronavirus were coded “1”, those who did not report an infection “0”. The distribution of self-infection across countries and our three pandemic phases is illustrated in the online appendix (Fig. AF6). Logistic regression results clearly indicate that neither openness to experience, agreeableness nor neuroticism influences how exposed one actually is to the Covid-19 infection risk (see Fig. 6). However, we find some evidence that extraverts are more exposed to the pandemic hazard (four coefficients p < 0.05; two p < 0.1). This relationship is most visible in Italy and Spain during the severe phases of the crisis (spring 2020 and winter 2020/2021). Moreover, people scoring high on conscientiousness are less likely to be exposed to infection (seven coefficients p < 0.05 and three p < 0.01). This holds in particular for Spain and Italy.

### Question 4: Who Favors the Closure of International Borders to Contain the Pandemic?

**Fig. 7:**
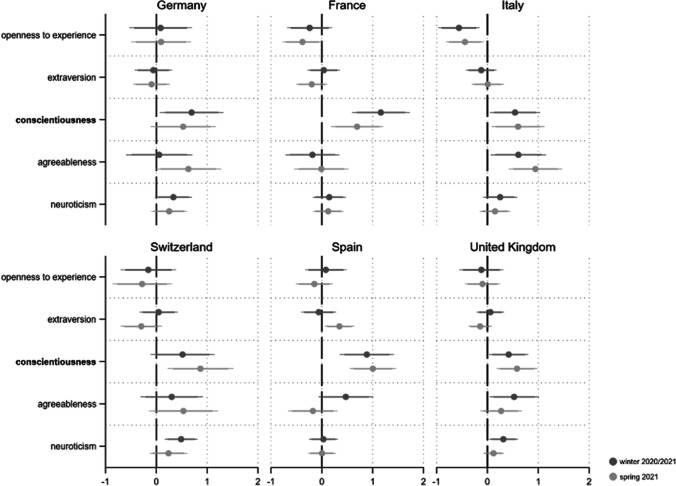
Personality and perceived importance of closing borders*. Note*: Displayed are linear regression coefficients (markers) with their 95 and 90% confidence intervals (horizontal lines). Personality traits with relatively robust effects are in bold. Models (fully presented in AT10a/b) control for gender, age, education and income situation

To evaluate the relationship between personality traits and political measures to contain the pandemic we rely on the perceived importance of the closing of international borders (1 “completely unimportant” to 7 “extremely important”; not surveyed in spring 2020) (see Fig. AF7 in the online appendix for an overview of the perceived importance of border closure across countries in winter 2020/2021 and spring 2021). The empirical analyses demonstrate that people scoring high on conscientiousness assess the closure of borders as important, whereas more unreliable individuals are much less convinced of this political measure to contain the pandemic (see Fig. 7). Of the 12 estimated coefficients for this relationship, 10 are statistically significant (p < 0.05). Thus, the effect of conscientiousness is highly robust across both countries and pandemic phases. We further find five positive and significant coefficients for agreeableness (three of them p < 0.05, two of them p < 0.1), providing some evidence that this trait is also related to a higher perceived importance of closing borders in a moderately robust way. This does not hold for France and Switzerland however. As for neuroticism, extraversion and openness to experience, we find few significant relationships. However, at least in Italy (and to a lesser extent also in France), open individuals seem to oppose the closure of international borders.

### Question 5: Who Tolerates Deviant Behavior Associated with Covid-19?

We define ‘tolerance’ as the willingness to allow “ideas and opinions that one dislikes or disagrees with” (Orlenius, 2008, p. 469). We measure Covid-19-related (in-)tolerance based on an adapted instrument used in the General Social Survey (The General Social Survey (GSS*)*, 2021) and asked respondents whether they would mind if someone who ignores non-pharmaceutical measures against the spread of Coronavirus (e.g., social distancing, mask-wearing, or quarantine requirements) would a) hold public office, be b) their boss, c) their neighbor, or d) a teacher (1 “yes”, 0 “no”) (cf. Schafer & Shaw, 2009, p. 415ff.). An overview of the respective tolerance levels across the six countries and the three pandemic phases is provided in the online appendix (Fig. AF8).

**Fig. 8:**
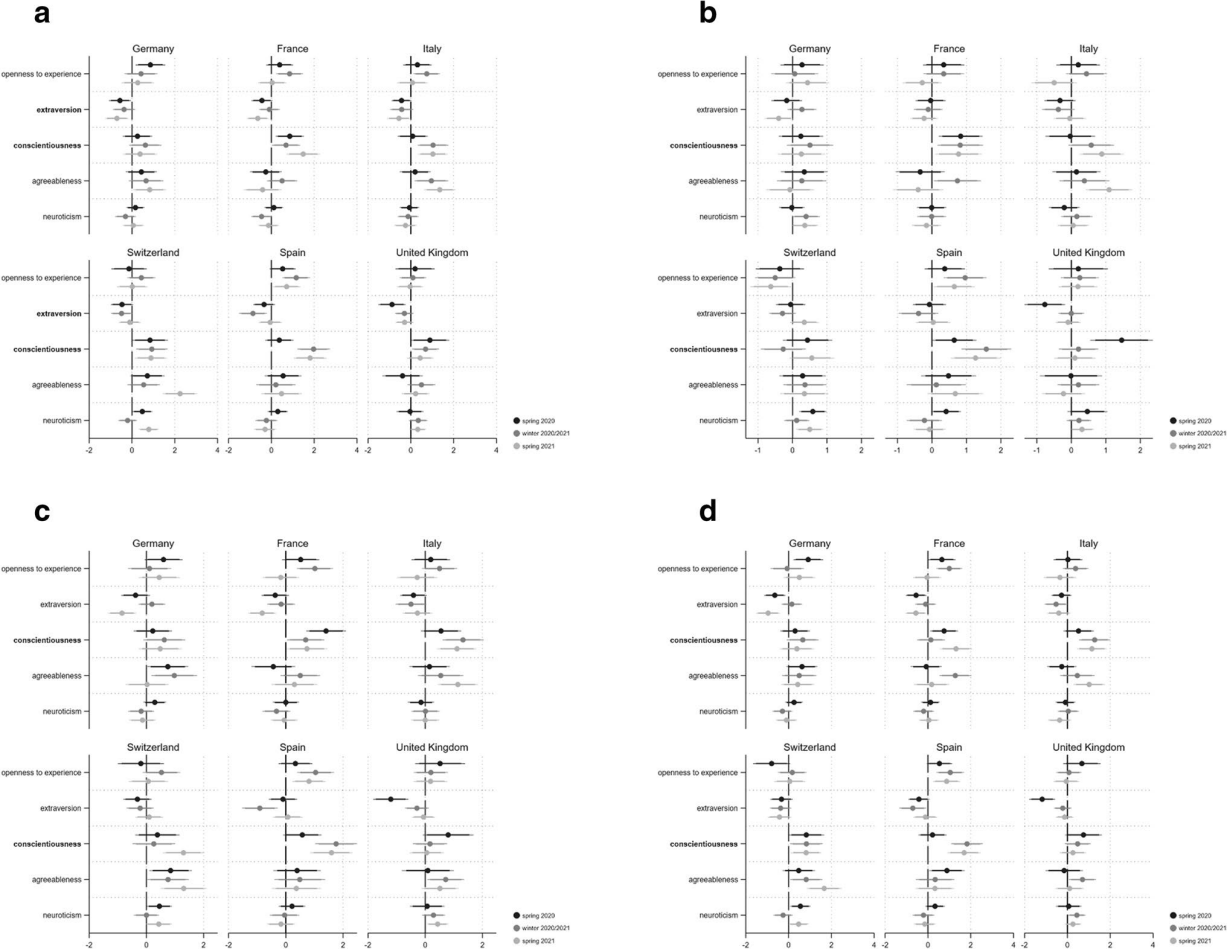
**a** Personality and Covid-19 Intolerance: Public Office Holder*. Note*: Displayed are logistic regression coefficients (markers) with their 95 and 90% confidence intervals (horizontal lines). Personality traits with relatively robust effects are in bold. Models (fully presented in AT11a/b) control for gender, age, education and income situation. **b** Personality and Covid-19 Intolerance: Neighbor. : Displayed are logistic regression coefficients (markers) with their 95 and 90% confidence intervals (horizontal lines). Personality traits with relatively robust effects are in bold. Models (fully presented in AT12a/b) control for gender, age, education and income situation. **c** Personality and Covid-19 Intolerance: Boss. : Displayed are logistic regression coefficients (markers) with their 95 and 90% confidence intervals (horizontal lines). Personality traits with relatively robust effects are in bold. Models (fully presented in AT13a/b) control for gender, age, education and income situation. **d** Personality and Covid-19 Intolerance: Teacher. *Note*: Displayed are logistic regression coefficients (markers) with their 95 and 90% confidence intervals (horizontal lines). Personality traits with relatively robust effects are in bold. Models (fully presented in AT14a/b) control for gender, age, education and income situation

We first look at the acceptance of persons with epidemiologically undesirable behavior as holders of public office (see Fig. 8a). For this form of tolerance, conscientiousness and extraversion in particular are decisive. While the former trait increases the probability of rejecting potential officials ignoring measures against the spread of Coronavirus, extraversion is related to tolerating deviant behavior. For conscientiousness, two thirds of the 18 coefficients are statistically significant (11 coefficients p < 0.05, one p < 0.1).

While we find no such effect in Germany, it is quite robust in France and Switzerland. With respect to extraversion, six coefficients show p < 0.05 and four p < 0.1. Throughout the pandemic, we find only very few indications that openness to experience, agreeableness and neuroticism are related to this kind of Covid-19 intolerance. Regarding the alternative measures of Covid-19 related intolerance, people scoring high on conscientiousness are not willing to accept someone as a neighbor if that person has shown epidemiologically undesirable behavior (especially in Spain and France, see Fig. 8b). Overall, 10 out of 18 coefficients are significant (eight p < 0.05 and two p < 0.1). We find a similar result for people with high scores for neuroticism (but only in six out of 18 coefficients, two p < 0.05, four p < 0.1). Conscientious individuals also reject ‘Covid-19-rulebreakers’ as their bosses in a relatively systematic and robust manner (Fig. 8c). Here, 11 out of 18 coefficients indicate a systematic effect (eight p < 0.05, three p < 0.1). Again this is most clearly shown in France and Spain. Moreover, people with high values for agreeableness do not seem to tolerate people showing epidemiologically undesirable behavior as their bosses (seven coefficients p < 0.05). This relationship holds particularly true for Switzerland but not for France and Spain. Finally, with regard to Fig. 8d, conscious people do not want teachers working if they do not comply with the non-pharmaceutical measures against the spread of Coronavirus (eight coefficients p < 0.05, three p < 0.1).

This is especially evident in Switzerland. In contrast, extraverts tolerate people working as teachers, even if they show this kind of deviant behavior (six coefficients p < 0.05 and two p < 0.1).

## The Pandemic Personality: Summary and Discussion

Summing up our empirical findings on the consequences of personality differences on Covid-19-related outcomes, we arrive at the following conclusions: First, regarding neuroticism, we empirically substantiate that emotionally unstable individuals feel particularly threatened by Covid-19, whether in terms of health, finances or social relationships. The respective empirical relationships are highly stable across the three pandemic phases and six countries under study. In addition, people scoring high on neuroticism consequently experience the negatively bearing emotions of fear and anger. While relevant for the mental states during the pandemic, we find little evidence that neuroticism is also related to pandemic exposure or social and political preferences in light of Covid-19.

Second, conscientiousness emerges as a salient trait in the context of the current pandemic, especially when it comes to protection against the virus. In more than half our models we find conscientious individuals to be less prone to infection with Coronavirus. What is more, people scoring high on this trait consistently perceive the closure of international borders as more important to fight the pandemic, indicating that they prefer collective security at the expense of individual liberties. Finally, conscientious people clearly do not tolerate people showing epidemiologically undesirable behavior as their bosses, neighbors, teachers or as public office holders, especially in France, Italy and Spain.

Third, most likely because of their social and gregarious nature, extroverts tend to perceive Covid-19 as a stronger threat to their social relationships. We also find tentative evidence that people scoring high on extraversion are more exposed to the risk of infection. With regard to Covid-19 intolerance, there are some indications that extraversion is related to a toleration of people who show deviant behavior. However, these relationships do not occur in every country and pandemic phase.

Fourth, we find little evidence that openness to experience plays a vital role in times of a pandemic. Openness neither robustly affects Covid-19 threat perception, emotional reactions to the pandemic, nor how exposed someone is to the pandemic hazard. Only the results for Italy and France reveal moderate evidence that open-minded people oppose closing borders to contain the pandemic. While open individuals are lauded for their high tolerance in other research contexts, we cannot report any robust relationship between this trait and Covid-19 intolerance.

Finally, as for openness, we find little evidence that agreeableness is a decisive trait in times of the pandemic. Regarding perceived threats from Covid-19, pandemic exposure, political and social preferences, agreeableness is not a clear and consistent foundation. However, agreeable individuals are not plagued by feelings of anger; instead, it is the more quarrelsome, critical and uncompromising who feel upset and hostile during the pandemic.

## Conclusion

How to explain the variety and diversity of behavioral, cognitive and psychological reactions to the current global health crisis triggered by Covid-19? In this paper, we explore whether personal dispositions can help explain the different ways of responding to the current pandemic. Focusing on the consequences of personality differences on hitherto neglected variables such as pandemic exposure, different emotional reactions and social and political preferences, we provide evidence regarding the function and value of the Big Five framework in understanding the pandemic personality in six countries that were among the most severely affected in Europe at the onset of the crisis. Using data of more than 18,000 respondents from Italy, Germany, Spain, the United Kingdom and Switzerland, polled during three pivotal phases of the Coronavirus pandemic (spring 2020, winter 2020/2021, and spring 2021), we significantly expand our understanding of the personality psychology imprint of individual pandemic experiences and responses.

Summing up our empirical insights, we arrive at the following conclusions: First, neuroticism is linked to mental states during the pandemic. Like no other trait, we find emotional instability to relate to heightened perceptions of health, financial and social threat as well as to higher levels of fear and anger throughout the Covid-19 crisis and across the six countries under study. Second, we find evidence that conscientiousness is most important when it comes to protection against the virus. Conscientiousness lowers infection risk, promotes intolerance towards epidemiologically undesirable behavior and is positively linked to a higher perceived necessity to close international borders as a political measure to contain the pandemic. All other Big Five personality traits show less robust effects. At most, it should be noted that extraversion tends to make infection more likely and seems to foster tolerance of nonconforming behavior in times of pandemic. Agreeableness leads to less anger in dealing with the pandemic and finally, openness to experience does not matter much during the times of Covid-19.

It has to be noted, however, that we find most personality effects to vary considerably across countries and pandemic development. This underlines the importance of examining such effects in a truly comparative manner, i.e. examining them cross-contextually and *separately* across the different countries and time points. For an analysis operating at the individual level, we think this is a sound way to make valid statements about the generalizability or robustness of personality effects.

Our findings have rich implications for public health politics, policy-makers and social cohesion. For example, in each case, a (large) majority of respondents in our datasets describe themselves as conscientious. This implies that a clear majority of West Europeans are predisposed to consider the closure of borders to be very important in fighting the pandemic. Furthermore, we find surprisingly high levels of intolerance towards people who ignore rules relevant for Covid-19-containment. Again, conscientiousness in particular promotes this kind of intolerance, whereas extraversion tends to be associated with tolerance of deviant behavior. Thus, West European societies see themselves confronted with high proportions of people who are, due to their personality, inclined to reject deviant behavior associated with Covid-19. While this implies a relatively high level of social control – and thus may be beneficial for pandemic containment – it also indicates a high potential for social conflict which could significantly challenge societal solidarity and cohesion in times of a pandemic and its aftermath. We further substantiate that neurotics in particular are mentally or psychologically challenged by the pandemic and thus in special need of (emotional) support to be able to cope well with it. Official contact and information points, hotlines or counselling services that directly address such people and assist them are possible ways to use our findings to the benefit of this certainly rather small but very vulnerable group. Finally, regarding our findings on pandemic exposure, public health authorities and policy makers can use our results to directly target the susceptible groups of people scoring low on conscientiousness (and high on extraversion) by taking into account their patterns of thoughts, feelings, and behavior (cf. Michels et al., 2021).

Yet, our study also has its limitations that require further attention. First, as we use cross-sectional data, strictly speaking, we cannot make causal claims. It has to be noted, however, that the genetic anchoring of personality and its high stability over the life course, both proven by previous research, support the causal link of the relationship that we have assumed (McCrae & Costa, 2008; Mondak, 2010; Stapleton et al., 2021). Furthermore, next to some cross-nationally robust relationships, we also report variable personality effects on Covid-19-relevant outcomes. However, no clear overarching pattern referring to pandemic phases, countries’ pandemic affectedness or policies can be identified to explain this variance. Accordingly, the impact of personality effects across different situations of Covid-19 presents a promising venue for future research, be it through the addition of further countries or through the analysis of regional entities. To this end, multi-level analyses considering the interplay between contextual factors and personality traits would be informative. What is more, while we found some robust relationships in our six-country set-up, studies in other cultural contexts have yet to prove the generalizability of these outside Western Europe. In addition, it should be noted that we evaluate the effects of the Big Five personality traits on Covid-19 relevant outcomes in an additive manner. Subsequent research could instead conceptualize personality on the basis of personality types and thus elicit the influence of personality as a product of individual traits (cf. Specht et al., 2014). Finally, future studies could provide valuable insights into personality’s imprint on even more Covid-19-relevant outcomes such as the assessment of vaccination campaigns, the public’s trust in (different) vaccinations and their affective reactions to them. In the threatening and uncertainty-inducing setting of the current pandemic, personality does play a vital role in shaping our behavior, feeling and thinking. We pass this finding on to the relevant research, hoping it will stimulate further interesting insights into the pandemic personality across different contexts.

## Supplementary Information

ESM 1 (DOCX 836 KB)

## Data Availability

The datasets generated during and/or analyzed during the current study are available from the corresponding author on reasonable request.

## Footnotes

1 However, Aschwanden et al. (2021) find higher neuroticism associated with fewer precautions and unrelated to preparatory behaviors related to Covid-19. Brouard et al. (2020) were surprised to find a negative association between neuroticism and compliance with the Covid-19 public health measures in France once emotions were taken into account.

2 We are, of course, aware that the pandemic did not proceed in exactly the same way in all countries during the respective periods and that our classification of the three pandemic phases can only refer to a very general European trend. For the first two phases, this overall trend is illustrated, for example, by the New York Times, which uses average deaths and hospitalizations in Europe to speak of a spring wave (peak: April 9, 2020) and an autumn wave (peak: November 28, 2020) (Holder et al., 2020). The sharp increase in the share of people fully vaccinated in the countries under study during spring 2021 is visible, for example, in Our World in Data *(*2021).

3 On the basis of all our subsamples, a total of 180 reliability indicators could be estimated (36 per personality trait, a total of 90 Cronbach alpha and 90 Spearman Brown estimates). In some survey waves and countries openness to experience and agreeableness especially show lower internal consistency values. However, following the relevant literature, difficulties in the measurement reliability of these two personality dimensions occur frequently (John et al., 2008, p. 131; Mondak et al., 2010, p. 78). The 180 reliability estimates are available on request.

4 In general, ordered logistic models should fulfil the parallel regression assumption. However, Brant-tests reveal that this assumption is violated in several of our models. Generalized ordered logit models (GOLMs) (Williams, 2005) did not substantially change our results and conclusions. Accordingly, to simplify interpretation, we decided to present the ordered logistic models to discuss our findings. Findings from the GOLMs are available upon request.

5 In the United Kingdom, the effect of conscientiousness on perceived financial threat during the second Corona wave (winter 2020/21) is even significantly negative (p < 0.05).

6 Anger is located within an emotional cluster called aversion (Brader & Marcus, 2013). The terms “aversion” and “anger” are sometimes used interchangeably in the relevant literature, i.e., naming the same appraisal dimension. Whether one understands the two as synonymous or sees anger as part of the broader concept of aversion, the underlying neural process is the same (Marcus et al., 2019, p. 121). According to Affective Intelligence Theory (Marcus et al., 2000), fear and aversion are situated in different parts of the brain that are connected with varying degrees to approach or avoidance behavior (Carver & White, 1994; Huddy et al., 2007, p. 211). While aversion signals that a threat is harmful to familiar norms and practices of thought, anxiety (fear) signifies the extent to which the threat is novel or uncertain.

7 Conducting confirmatory factor analyses (CFA) for each country, our results indicate that the two-factor solution with one factor for fear and another for anger is superior to a one-factor solution including all items. Results are available upon request.

8 As neuroticism per definition is related to negative emotional experiences and feelings of anxiety, we further tested whether our results also hold for explicitly Covid-19-induced fear and anger (surveyed in winter 2020/2021 and spring 2021). The originally stimuli-independent PANAS-scale was slightly adapted by asking respondents to indicate how often in the last weeks and months they have felt a list of different emotions and feelings in relation to a possible infection with Coronavirus (ranging from 1 “never” to 5 “very often”). To measure Covid-19-induced fear we combined the frequencies of feeling “anxious” and “worried”, while for anger, the affective terms “upset” and “hostile” were used. Regarding fear, again all coefficients for neuroticism pointed in the expected direction and were statistically significant at the 0.05 level. This is also true with regard to anger. What is more, the negative relationship of agreeableness with anger was further supported (ten coefficients p < 0.05, one coefficient with p < 0.1) Full results from these analyses are available upon request.
