# Supplementary material for: Pandemic personality: Emotional reactions, political and social preferences across personality traits in times of Corona
Source: Curr Psychol. 2021 Nov 23:1–15. Online ahead of print. doi: 10.1007/s12144-021-02493-x (PMC8610108; doi:10.1007/s12144-021-02493-x)
Supplement: Supplementary file 1 — (DOCX 836 KB) [file 12144_2021_2493_MOESM1_ESM.docx]

**Online Appendix:**

**“Pandemic personality: Emotional reactions, political and social preferences across personality traits in times of Corona” (Authors: Markus Freitag and Nathalie Hofstetter, published in: Current Psychology)**

**AT0**: Description of the surveys

|  | Spring 2020 | Winter 2020/2021 | Spring 2021 |
| --- | --- | --- | --- |
| Survey period | April 17, 2020 to May 11, 2020 | November 24, 2020 to January 18, 2021 | April 22, 2021 to May 21, 2021 |
|  |  |  |  |
| Target population | Residents aged 18 years or older in Germany, France, Italy, Switzerland, Spain and the United Kingdom | Residents aged 18 years or older in Germany, France, Italy, Switzerland, Spain and the United Kingdom | Residents aged 18 years or older in Germany, France, Italy, Switzerland, Spain and the United Kingdom |
|  |  |  |  |
| Survey mode | Online | Online | Online |
|  |  |  |  |
| Sample size | 6,028 respondents (target sample size: 1,000 per country) | 6,210 respondents (target sample size: 1,000 per country) | 6,069 respondents (target sample size: 1,000 per country) |
|  |  |  |  |
| Quotas | Age, Sex, Education (language for Switzerland) | Age, Sex, Education (language for Switzerland) | Age, Sex, Education (language for Switzerland) |
|  |  |  |  |
| Sampling | Qualtrics panel | Survey Engine panel(s) | Survey Engine panel(s) |
|  |  |  |  |
| Interview language | German, French, Italian, Spanish, English | German, French, Italian, Spanish, English | German, French, Italian, Spanish, English |
|  |  |  |  |
| Response rate | Overall: 8.71% | Overall: 7.03% | Overall: 17.86% |
|  |  |  |  |
| Institute | survey carried out by Qualtrics | survey carried out by Survey Engine | survey carried out by Survey Engine |
|  |  |  |  |

**AT1**: Descriptive Overview: The Big Five Personality Traits

|  | Spring 2020 | Winter 2020/2021 | Spring 2021 | Total |
| --- | --- | --- | --- | --- |
|  |  |  |  |  |
| Share of respondents scoring high on… |  |  |  |  |
|  |  |  |  |  |
| …Openness to  experience | 30.7 | 34.1 | 35.2 | 33.4 |
|  |  |  |  |  |
| …Conscientiousness | 59.3 | 63.0 | 64.3 | 62.2 |
|  |  |  |  |  |
| …Extraversion | 13.1 | 15.1 | 15.2 | 14.5 |
|  |  |  |  |  |
| …Agreeableness | 40.6 | 43.6 | 45.1 | 43.1 |
|  |  |  |  |  |
| …Neuroticism | 8.5 | 9.1 | 9.1 | 8.9 |
|  |  |  |  |  |
| Observations | 6,028 | 6,210 | 6,069 | 18,307 |
|  |  |  |  |  |

*Note*: The proportions displayed equal the share of respondents who achieve a value of at least 4 on the arithmetic index [1:5]

for the respective personality dimension in each pandemic phase and in a combined dataset that includes the observations

from all three pandemic phases.

**AT2**: Descriptive Overview: The Big Five Personality Traits Across Countries

|  | Germany | France | Italy | Switzerland | Spain | United Kingdom |
| --- | --- | --- | --- | --- | --- | --- |
|  |  |  |  |  |  |  |
| Share of respondents scoring high on… |  |  |  |  |  |  |
|  |  |  |  |  |  |  |
| …Openness to  experience | S1: 45.3  S2: 47.5  S3: 46.4  Total: 46.4 | S1: 25.6  S2: 27.0  S3: 30.1  Total: 27.6 | S1: 14.8  S2: 15.1  S3: 16.9  Total: 15.6 | S1: 46.2  S2: 44.8  S3: 44.3  Total: 45.1 | S1: 35.9  S2: 32.3  S3: 34.9  Total: 34.4 | S1: 16.3  S2: 37.0  S3: 38.7  Total: 30.7 |
|  |  |  |  |  |  |  |
| …Conscientiousness | S1: 66.5  S2: 71.3  S3: 71.3  Total: 69.7 | S1: 64.0  S2: 66.1  S3: 66.8  Total: 65.6 | S1: 59.3  S2: 54.8  S3: 58.3  Total: 57.5 | S1: 68.6  S2: 69.2  S3: 69.6  Total: 69.2 | S1: 58.7  S2: 56.2  S3: 56.7  Total: 57.2 | S1: 38.4  S2: 59.9  S3: 63.4  Total: 53.9 |
|  |  |  |  |  |  |  |
| …Extraversion | S1: 19.7  S2: 20.3  S3: 18.8  Total: 19.6 | S1: 9.6  S2: 9.6  S3: 9.5  Total: 9.6 | S1: 11.6  S2: 13.4  S3: 13.5  Total: 12.8 | S1: 17.4  S2: 18.3  S3: 17.9  Total: 17.9 | S1: 14.7  S2: 11.9  S3: 13.9  Total: 13.5 | S1: 5.8  S2: 16.8  S3: 17.9  Total: 13.5 |
|  |  |  |  |  |  |  |
| …Agreeableness | S1: 48.6  S2: 50.2  S3: 51.0  Total: 50.0 | S1: 48.1  S2: 47.7  S3: 49.1  Total: 48.3 | S1: 48.3  S2: 46.2  S3: 50.4  Total: 48.3 | S1: 53.6  S2: 52.8  S3: 53.7  Total: 53.4 | S1: 17.3  S2: 19.4  S3: 19.7  Total: 18.8 | S1: 27.3  S2: 44.5  S3: 47.8  Total: 39.9 |
|  |  |  |  |  |  |  |
| …Neuroticism | S1: 7.7  S2: 8.0  S3: 7.8  Total: 7.8 | S1: 11.1  S2: 9.4  S3: 10.5  Total: 10.3 | S1: 11.0  S2: 9.6  S3: 9.2  Total: 9.9 | S1: 6.3  S2: 6.3  S3: 7.3  Total: 6.6 | S1: 7.8  S2: 7.8  S3: 7.0  Total: 7.5 | S1: 7.0  S2: 13.9  S3: 13.3  Total: 11.4 |
|  |  |  |  |  |  |  |
| Observations | S1: 1,006  S2: 1,000  S3: 1,007  Total: 3,013 | S1: 1,010  S2: 1,030  S3: 1,005  Total: 3,045 | S1: 1,001  S2: 1,000  S3: 1,004  Total: 3,005 | S1: 1,005  S2: 1,128  S3: 1,010  Total: 3,143 | S1: 1,000  S2: 1,013  S3: 1,040  Total: 3,053 | S1: 1,006  S2: 1,039  S3: 1,003  Total: 3,048 |
|  |  |  |  |  |  |  |

*Note*: The proportions displayed equal the share of respondents per country who achieve a value of at least 4 on the arithmetic index [1:5] for the respective personality

dimension in each pandemic phase (S1 = spring 2020; S2 = winter 2020/2021; S3 = spring 2021) and in a combined dataset that includes the observations from all three

pandemic phases.

**AT3**: Descriptive Overview: Control Variables

|  | **N** | | | **Mean** | | | **SD** | | | **[Min:Max]** | | |
| --- | --- | --- | --- | --- | --- | --- | --- | --- | --- | --- | --- | --- |
| Pandemic phase | Spring 2020 | Winter 2020/21 | Spring 2021 | Spring 2020 | Winter 2020/21 | Spring 2021 | Spring 2020 | Winter 2020/21 | Spring 2021 | Spring 2020 | Winter 2020/21 | Spring 2021 |
|  |  |  |  |  |  |  |  |  |  |  |  |  |
| **Germany** |  |  |  |  |  |  |  |  |  |  |  |  |
| Education |  |  |  |  |  |  |  |  |  |  |  |  |
| Upper secondary | 1,006 | 1,000 | 1,007 | 0.57 | 0.57 | 0.57 | 0.50 | 0.50 | 0.50 | [0:1] | [0:1] | [0:1] |
| Tertiary | 1,006 | 1,000 | 1,007 | 0.29 | 0.29 | 0.29 | 0.45 | 0.45 | 0.46 | [0:1] | [0:1] | [0:1] |
|  |  |  |  |  |  |  |  |  |  |  |  |  |
| Male | 1,006 | 1,000 | 1,007 | 0.50 | 0.50 | 0.50 | 0.50 | 0.50 | 0.50 | [0:1] | [0:1] | [0:1] |
|  |  |  |  |  |  |  |  |  |  |  |  |  |
| Age | 1,006 | 1,000 | 1,007 | 48.84 | 48.14 | 48.22 | 16.19 | 16.82 | 16.49 | [18:82] | [18:87] | [18:88] |
|  |  |  |  |  |  |  |  |  |  |  |  |  |
| Income situation | 1,003 | 989 | 990 | 3.09 | 3.18 | 3.21 | 1.05 | 1.04 | 1.04 | [1:5] | [1:5] | [1:5] |
|  |  |  |  |  |  |  |  |  |  |  |  |  |
| **France** |  |  |  |  |  |  |  |  |  |  |  |  |
| Education |  |  |  |  |  |  |  |  |  |  |  |  |
| Upper secondary | 1,010 | 1,030 | 1,005 | 0.43 | 0.42 | 0.43 | 0.49 | 0.49 | 0.50 | [0:1] | [0:1] | [0:1] |
| Tertiary | 1,010 | 1,030 | 1,005 | 0.38 | 0.37 | 0.38 | 0.48 | 0.48 | 0.48 | [0:1] | [0:1] | [0:1] |
|  |  |  |  |  |  |  |  |  |  |  |  |  |
| Male | 1,010 | 1,030 | 1,005 | 0.50 | 0.50 | 0.50 | 0.50 | 0.50 | 0.50 | [0:1] | [0:1] | [0:1] |
|  |  |  |  |  |  |  |  |  |  |  |  |  |
| Age | 1,010 | 1,030 | 1,005 | 49.07 | 47.96 | 47.57 | 16.01 | 16.50 | 15.93 | [18:83] | [18:91] | [18:90] |
|  |  |  |  |  |  |  |  |  |  |  |  |  |
| Income situation | 1,007 | 1,022 | 983 | 2.86 | 2.88 | 2.92 | 1.03 | 1.02 | 0.99 | [1:5] | [1:5] | [1:5] |
|  |  |  |  |  |  |  |  |  |  |  |  |  |
| **Italy** |  |  |  |  |  |  |  |  |  |  |  |  |
| Education |  |  |  |  |  |  |  |  |  |  |  |  |
| Upper secondary | 1,001 | 1,000 | 1,004 | 0.43 | 0.42 | 0.47 | 0.50 | 0.49 | 0.50 | [0:1] | [0:1] | [0:1] |
| Tertiary | 1,001 | 1,000 | 1,004 | 0.20 | 0.19 | 0.26 | 0.40 | 0.39 | 0.44 | [0:1] | [0:1] | [0:1] |
|  |  |  |  |  |  |  |  |  |  |  |  |  |
| Male | 1,001 | 1,000 | 1,004 | 0.51 | 0.50 | 0.49 | 0.50 | 0.50 | 0.50 | [0:1] | [0:1] | [0:1] |
|  |  |  |  |  |  |  |  |  |  |  |  |  |
| Age | 1,001 | 1,000 | 1,004 | 48.46 | 47.72 | 48.71 | 16.59 | 16.29 | 15.34 | [18:87] | [18:89] | [18:86] |
|  |  |  |  |  |  |  |  |  |  |  |  |  |
| Income situation | 993 | 981 | 977 | 2.63 | 2.66 | 2.81 | 1.01 | 1.03 | 1.04 | [1:5] | [1:5] | [1:5] |
|  |  |  |  |  |  |  |  |  |  |  |  |  |
| **Switzerland** |  |  |  |  |  |  |  |  |  |  |  |  |
| Education |  |  |  |  |  |  |  |  |  |  |  |  |
| Upper secondary | 1,005 | 1,128 | 1,010 | 0.46 | 0.46 | 0.47 | 0.50 | 0.50 | 0.50 | [0:1] | [0:1] | [0:1] |
| Tertiary | 1,005 | 1,128 | 1,010 | 0.45 | 0.44 | 0.46 | 0.50 | 0.50 | 0.50 | [0:1] | [0:1] | [0:1] |
|  |  |  |  |  |  |  |  |  |  |  |  |  |
| Male | 1,005 | 1,128 | 1,010 | 0.50 | 0.50 | 0.51 | 0.50 | 0.50 | 0.50 | [0:1] | [0:1] | [0:1] |
|  |  |  |  |  |  |  |  |  |  |  |  |  |
| Age | 1,005 | 1,128 | 1,010 | 49.16 | 48.30 | 48.55 | 16.99 | 17.17 | 16.72 | [18:85] | [18:86] | [18:86] |
|  |  |  |  |  |  |  |  |  |  |  |  |  |
| Income situation | 990 | 1,120 | 990 | 3.09 | 3.11 | 3.14 | 1.09 | 1.11 | 1.07 | [1:5] | [1:5] | [1:5] |
|  |  |  |  |  |  |  |  |  |  |  |  |  |
| **Spain** |  |  |  |  |  |  |  |  |  |  |  |  |
| Education |  |  |  |  |  |  |  |  |  |  |  |  |
| Upper secondary | 1,000 | 1,013 | 1,040 | 0.24 | 0.25 | 0.29 | 0.43 | 0.43 | 0.54 | [0:1] | [0:1] | [0:1] |
| Tertiary | 1,000 | 1,013 | 1,040 | 0.38 | 0.38 | 0.41 | 0.49 | 0.49 | 0.49 | [0:1] | [0:1] | [0:1] |
|  |  |  |  |  |  |  |  |  |  |  |  |  |
| Male | 1,000 | 1,013 | 1,040 | 0.51 | 0.50 | 0.52 | 0.50 | 0.50 | 0.50 | [0:1] | [0:1] | [0:1] |
|  |  |  |  |  |  |  |  |  |  |  |  |  |
| Age | 1,000 | 1,013 | 1,040 | 48.00 | 48.00 | 47.27 | 16.09 | 14.88 | 15.20 | [18:88] | [18:88] | [18:87] |
|  |  |  |  |  |  |  |  |  |  |  |  |  |
| Income situation | 992 | 1,002 | 1,016 | 2.66 | 2.84 | 2.92 | 1.08 | 1.10 | 1.04 | [1:5] | [1:5] | [1:5] |
|  |  |  |  |  |  |  |  |  |  |  |  |  |
| **United Kingdom** |  |  |  |  |  |  |  |  |  |  |  |  |
| Education |  |  |  |  |  |  |  |  |  |  |  |  |
| Upper secondary | 1,006 | 1,039 | 1,003 | 0.21 | 0.21 | 0.23 | 0.41 | 0.41 | 0.42 | [0:1] | [0:1] | [0:1] |
| Tertiary | 1,006 | 1,039 | 1,003 | 0.46 | 0.46 | 0.47 | 0.50 | 0.50 | 0.50 | [0:1] | [0:1] | [0:1] |
|  |  |  |  |  |  |  |  |  |  |  |  |  |
| Male | 1,006 | 1,039 | 1,003 | 0.50 | 0.50 | 0.49 | 0.50 | 0.50 | 0.50 | [0:1] | [0:1] | [0:1] |
|  |  |  |  |  |  |  |  |  |  |  |  |  |
| Age | 1,006 | 1,039 | 1,003 | 47.26 | 46.60 | 46.72 | 17.34 | 17.12 | 17.31 | [18:84] | [18:87] | [18:87] |
|  |  |  |  |  |  |  |  |  |  |  |  |  |
| Income situation | 1,000 | 1,029 | 986 | 3.25 | 3.12 | 3.39 | 1.10 | 1.11 | 1.10 | [1:5] | [1:5] | [1:5] |
|  |  |  |  |  |  |  |  |  |  |  |  |  |

*Note*: Reference category for education is “primary and lower secondary”.

**AF1**: Descriptive Overview: Perception of Covid-19 as a (very) major health threat


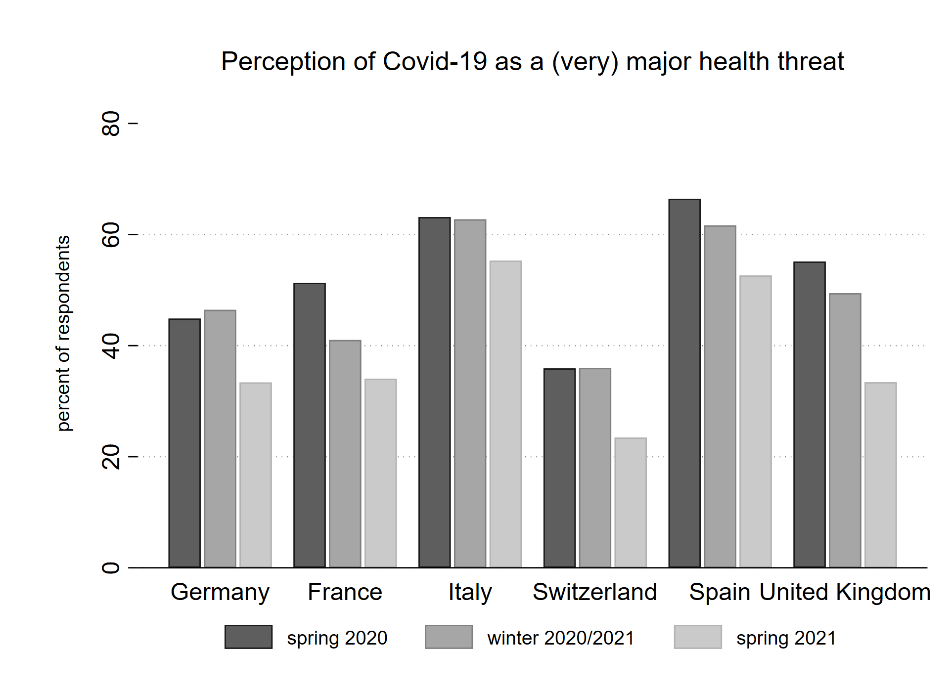


*Note*: The proportions displayed equal the share of respondents per country and

pandemic phase who perceive Covid-19 as a (very) major health threat.

**AT4a**: Big Five of Personality and Perceived Health Threat from Covid-19 in Germany, France and Italy

|  | **Germany** | | | **France** | | | **Italy** | | |
| --- | --- | --- | --- | --- | --- | --- | --- | --- | --- |
| Pandemic phase | Spring 2020 | Winter 2020/2021 | Spring 2021 | Spring 2020 | Winter 2020/2021 | Spring 2021 | Spring 2020 | Winter 2020/2021 | Spring 2021 |
|  |  |  |  |  |  |  |  |  |  |
| Openness to experience | 0.928^**^ (0.355) | 0.369 (0.409) | 0.527 (0.343) | -0.369 (0.311) | 0.443 (0.288) | -0.152 (0.365) | -0.152 (0.365) | -0.242 (0.397) | 0.250 (0.364) |
|  |  |  |  |  |  |  |  |  |  |
| Extraversion | 0.113 (0.205) | 0.276 (0.245) | -0.325 (0.261) | 0.254 (0.222) | 0.364 (0.231) | 0.154 (0.210) | 0.154 (0.210) | -0.398^*^ (0.241) | -0.368 (0.250) |
|  |  |  |  |  |  |  |  |  |  |
| Conscientiousness | 0.113 (0.348) | 0.207 (0.372) | 0.684 (0.481) | 0.418 (0.364) | -0.121 (0.380) | 0.092 (0.324) | 0.092 (0.324) | -0.046 (0.459) | 0.472 (0.407) |
|  |  |  |  |  |  |  |  |  |  |
| Agreeableness | 0.563^*^ (0.339) | -0.026 (0.406) | 0.430 (0.456) | 0.083 (0.360) | 0.292 (0.414) | 0.340 (0.355) | 0.340 (0.355) | 0.936^**^ (0.407) | -0.091 (0.453) |
|  |  |  |  |  |  |  |  |  |  |
| Neuroticism | 0.809^**^ (0.191) | 0.609^**^ (0.213) | 0.428^*^ (0.249) | 0.843^**^ (0.199) | 0.945^**^ (0.235) | 0.515^**^ (0.224) | 0.515^**^ (0.224) | 0.804^**^ (0.223) | 0.220 (0.254) |
|  |  |  |  |  |  |  |  |  |  |
| Education |  |  |  |  |  |  |  |  |  |
|  |  |  |  |  |  |  |  |  |  |
| Upper, post secondary | -0.242 (0.205) | -0.101 (0.202) | -0.161 (0.236) | 0.126 (0.177) | 0.290 (0.190) | -0.157 (0.150) | -0.157 (0.150) | -0.046 (0.167) | -0.188 (0.184) |
|  |  |  |  |  |  |  |  |  |  |
| Tertiary | 0.002 (0.217) | 0.374^*^ (0.221) | -0.035 (0.260) | 0.061 (0.204) | 0.237 (0.225) | 0.241 (0.192) | 0.241 (0.192) | 0.349^*^ (0.205) | 0.021 (0.226) |
|  |  |  |  |  |  |  |  |  |  |
| Male | -0.017 (0.135) | -0.455^**^ (0.150) | -0.053 (0.155) | -0.252^*^ (0.137) | -0.268^*^ (0.153) | -0.308^**^ (0.132) | -0.308^**^ (0.132) | -0.390^**^ (0.144) | -0.772^**^ (0.155) |
|  |  |  |  |  |  |  |  |  |  |
| Age | -0.003 (0.004) | 0.002 (0.004) | -0.006 (0.005) | -0.001 (0.005) | 0.004 (0.005) | 0.001 (0.004) | 0.001 (0.004) | 0.001 (0.005) | -0.010^*^ (0.006) |
|  |  |  |  |  |  |  |  |  |  |
| Income situation | -0.188^**^ (0.064) | -0.020 (0.074) | -0.189^**^ (0.087) | -0.067 (0.075) | -0.151^*^ (0.082) | -0.183^**^ (0.072) | -0.183^**^ (0.072) | -0.169^**^ (0.082) | -0.157^**^ (0.079) |
| / |  |  |  |  |  |  |  |  |  |
| cut1 | 0.015 (0.819) | -0.879 (1.029) | -0.505 (1.113) | -1.102 (0.792) | 0.047 (0.858) | -1.888^**^ (0.777) | -1.888^**^ (0.777) | -1.779^*^ (0.983) | -2.929^**^ (0.937) |
|  |  |  |  |  |  |  |  |  |  |
| cut2 | 2.124^**^ (0.823) | 1.461 (1.032) | 1.836^*^ (1.113) | 0.875 (0.786) | 2.144^**^ (0.859) | -0.186 (0.776) | -0.186 (0.776) | 0.062 (0.975) | -1.010 (0.923) |
|  |  |  |  |  |  |  |  |  |  |
| cut3 | 3.965^**^ (0.833) | 3.554^**^ (1.049) | 3.663^**^ (1.108) | 2.158^**^ (0.788) | 3.531^**^ (0.862) | 2.006^**^ (0.784) | 2.006^**^ (0.784) | 2.064^**^ (0.979) | 0.951 (0.927) |
| Pseudo *R*^2^ | 0.020 | 0.017 | 0.013 | 0.016 | 0.022 | 0.015 | 0.015 | 0.027 | 0.031 |
| Observations | 924 | 811 | 686 | 834 | 703 | 856 | 856 | 692 | 650 |

*Note*: Ordered logistic modeling coefficients with robust standard errors in parentheses. Reference category for education is “primary & lower secondary”. ^*^ *p* < 0.10, ^**^ *p* < 0.05

**AT4b**: Big Five of Personality and Perceived Health Threat from Covid-19 in Switzerland, Spain and the United Kingdom

|  | **Switzerland** | | | **Spain** | | | **United Kingdom** | | |
| --- | --- | --- | --- | --- | --- | --- | --- | --- | --- |
| Pandemic phase | Spring 2020 | Winter 2020/2021 | Spring 2021 | Spring 2020 | Winter 2020/2021 | Spring 2021 | Spring 2020 | Winter 2020/2021 | Spring 2021 |
|  |  |  |  |  |  |  |  |  |  |
| Openness to experience | -0.870^**^ (0.330) | -0.455 (0.366) | 0.187 (0.470) | -0.427 (0.310) | -0.733^**^ (0.369) | 0.123 (0.368) | -0.414 (0.435) | -0.123 (0.332) | -0.013 (0.324) |
|  |  |  |  |  |  |  |  |  |  |
| Extraversion | 0.200 (0.220) | -0.062 (0.257) | -0.352 (0.306) | 0.532^**^ (0.249) | 0.253 (0.290) | -0.084 (0.288) | 0.087 (0.286) | 0.075 (0.215) | -0.027 (0.225) |
|  |  |  |  |  |  |  |  |  |  |
| Conscientiousness | -0.268 (0.352) | 0.409 (0.467) | 0.307 (0.506) | 0.176 (0.322) | 0.712^*^ (0.413) | 1.017^**^ (0.408) | 1.707^**^ (0.392) | -0.202 (0.320) | 0.234 (0.336) |
|  |  |  |  |  |  |  |  |  |  |
| Agreeableness | 1.036^**^ (0.325) | 0.758 (0.483) | 0.923^*^ (0.473) | -0.134 (0.413) | -0.444 (0.449) | 0.004 (0.440) | 1.049^**^ (0.418) | 1.000^**^ (0.368) | 0.341 (0.360) |
|  |  |  |  |  |  |  |  |  |  |
| Neuroticism | 1.021^**^ (0.194) | 0.440^*^ (0.243) | 0.626^**^ (0.279) | 0.713^**^ (0.238) | 0.693^**^ (0.230) | 0.176 (0.258) | 1.545^**^ (0.287) | 0.699^**^ (0.217) | 0.592^**^ (0.201) |
|  |  |  |  |  |  |  |  |  |  |
| Education |  |  |  |  |  |  |  |  |  |
|  |  |  |  |  |  |  |  |  |  |
| Upper, post secondary | -0.040 (0.244) | -0.253 (0.276) | -0.377 (0.439) | 0.125 (0.181) | 0.063 (0.205) | -0.086 (0.228) | -0.355^*^ (0.183) | 0.320^*^ (0.183) | -0.131 (0.197) |
|  |  |  |  |  |  |  |  |  |  |
| Tertiary | 0.237 (0.253) | -0.110 (0.281) | -0.474 (0.445) | 0.387^**^ (0.171) | -0.174 (0.191) | -0.157 (0.221) | -0.224 (0.152) | 0.432^**^ (0.180) | -0.221 (0.174) |
|  |  |  |  |  |  |  |  |  |  |
| Male | -0.032 (0.139) | 0.014 (0.157) | 0.054 (0.173) | -0.181 (0.139) | -0.180 (0.156) | -0.507^**^ (0.165) | 0.158 (0.129) | -0.423^**^ (0.143) | -0.195 (0.154) |
|  |  |  |  |  |  |  |  |  |  |
| Age | -0.005 (0.004) | -0.010^**^ (0.005) | -0.018^**^ (0.005) | 0.005 (0.005) | 0.000 (0.005) | -0.007 (0.006) | -0.006 (0.004) | 0.009^*^ (0.005) | -0.007 (0.005) |
|  |  |  |  |  |  |  |  |  |  |
| Income situation | -0.143^**^ (0.071) | -0.177^**^ (0.075) | -0.240^**^ (0.091) | -0.066 (0.068) | 0.023 (0.073) | -0.174^*^ (0.090) | -0.209^**^ (0.066) | -0.172^**^ (0.062) | -0.214^**^ (0.076) |
| / |  |  |  |  |  |  |  |  |  |
| cut1 | -1.447^*^ (0.751) | -1.723 (1.108) | -1.206 (1.205) | -1.866^**^ (0.921) | -2.173^**^ (1.051) | -2.011^*^ (1.034) | 1.088 (0.988) | -0.535 (0.814) | -1.447 (0.884) |
|  |  |  |  |  |  |  |  |  |  |
| cut2 | 0.844 (0.748) | 0.649 (1.101) | 1.215 (1.193) | 0.186 (0.902) | -0.154 (1.041) | 0.249 (1.021) | 3.269^**^ (0.988) | 1.532^*^ (0.814) | 0.582 (0.885) |
|  |  |  |  |  |  |  |  |  |  |
| cut3 | 2.666^**^ (0.752) | 2.683^**^ (1.106) | 3.080^**^ (1.182) | 1.946^**^ (0.905) | 1.727^*^ (1.038) | 2.116^**^ (1.019) | 4.386^**^ (0.993) | 2.790^**^ (0.819) | 1.731^*^ (0.892) |
| Pseudo *R*^2^ | 0.031 | 0.016 | 0.036 | 0.015 | 0.016 | 0.020 | 0.030 | 0.026 | 0.022 |
| Observations | 866 | 673 | 554 | 738 | 631 | 552 | 897 | 808 | 727 |

*Note*: Ordered logistic modeling coefficients with robust standard errors in parentheses. Reference category for education is “primary & lower secondary”. ^*^ *p* < 0.10, ^**^ *p* < 0.05

**AF2**: Descriptive Overview: Perception of Covid-19 as a (very) major financial threat


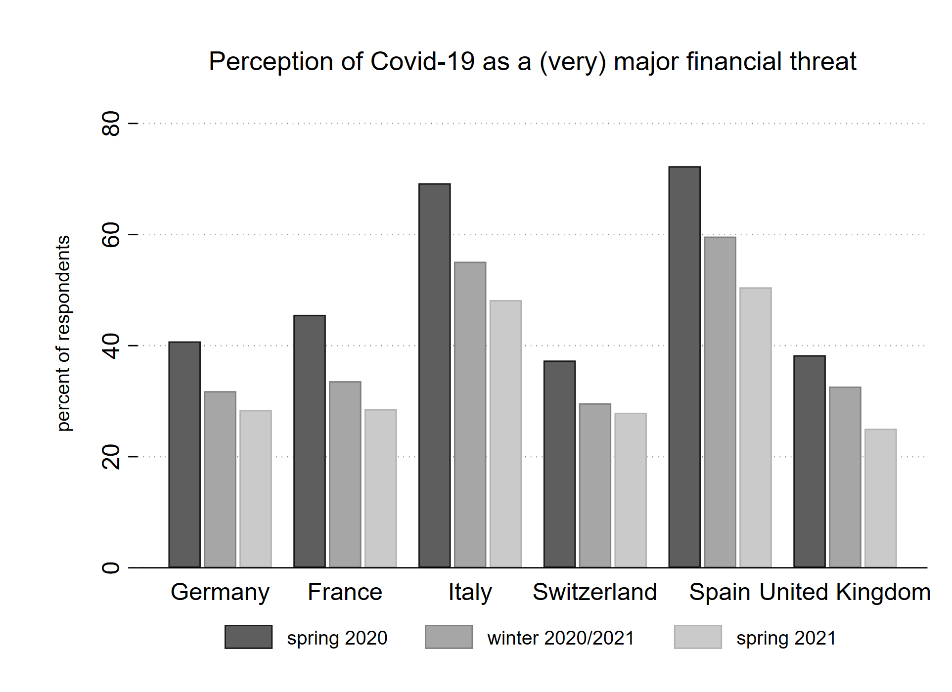


*Note*: The proportions displayed equal the share of respondents per country and

pandemic phase who perceive Covid-19 as a (very) major financial threat.

**AT5a**: Big Five of Personality and Perceived Financial Threat from Covid-19 in Germany, France and Italy

|  | **Germany** | | | **France** | | | **Italy** | | |
| --- | --- | --- | --- | --- | --- | --- | --- | --- | --- |
| Pandemic phase | Spring 2020 | Winter 2020/2021 | Spring 2021 | Spring 2020 | Winter 2020/2021 | Spring 2021 | Spring 2020 | Winter 2020/2021 | Spring 2021 |
|  |  |  |  |  |  |  |  |  |  |
| Openness to experience | 0.309 (0.323) | 0.443 (0.332) | 0.388 (0.306) | 0.147 (0.277) | 0.672^**^ (0.271) | 0.366 (0.280) | 0.150 (0.286) | 0.324 (0.264) | 0.366 (0.280) |
|  |  |  |  |  |  |  |  |  |  |
| Extraversion | 0.368^*^ (0.221) | -0.127 (0.203) | 0.224 (0.217) | 0.415^*^ (0.222) | 0.018 (0.187) | -0.036 (0.199) | -0.141 (0.198) | -0.446^**^ (0.190) | -0.036 (0.199) |
|  |  |  |  |  |  |  |  |  |  |
| Conscientiousness | -0.311 (0.301) | 0.553^*^ (0.331) | 0.976^**^ (0.327) | -0.057 (0.283) | -0.107 (0.329) | -0.179 (0.345) | -0.401 (0.328) | 0.430 (0.327) | -0.179 (0.345) |
|  |  |  |  |  |  |  |  |  |  |
| Agreeableness | 0.050 (0.324) | -0.259 (0.339) | -0.968^**^ (0.372) | -0.127 (0.302) | -0.531^*^ (0.314) | -0.609^**^ (0.289) | 0.237 (0.309) | -0.125 (0.302) | -0.609^**^ (0.289) |
|  |  |  |  |  |  |  |  |  |  |
| Neuroticism | 0.311^*^ (0.188) | 0.483^**^ (0.196) | 0.603^**^ (0.211) | 0.471^**^ (0.182) | 0.322 (0.198) | 0.366^*^ (0.208) | 0.241 (0.193) | 0.628^**^ (0.185) | 0.366^*^ (0.208) |
|  |  |  |  |  |  |  |  |  |  |
| Education |  |  |  |  |  |  |  |  |  |
|  |  |  |  |  |  |  |  |  |  |
| Upper, post secondary | 0.440^**^ (0.192) | 0.175 (0.194) | 0.100 (0.182) | 0.323^*^ (0.168) | -0.044 (0.164) | 0.159 (0.151) | 0.183 (0.140) | 0.219 (0.137) | 0.159 (0.151) |
|  |  |  |  |  |  |  |  |  |  |
| Tertiary | 0.564^**^ (0.212) | 0.064 (0.206) | 0.071 (0.202) | 0.276 (0.191) | 0.118 (0.182) | 0.357^*^ (0.191) | 0.431^**^ (0.176) | 0.294^*^ (0.173) | 0.357^*^ (0.191) |
|  |  |  |  |  |  |  |  |  |  |
| Male | -0.044 (0.126) | -0.204 (0.135) | -0.000 (0.135) | -0.161 (0.123) | -0.229^*^ (0.124) | -0.552^**^ (0.128) | -0.487^**^ (0.124) | -0.808^**^ (0.122) | -0.552^**^ (0.128) |
|  |  |  |  |  |  |  |  |  |  |
| Age | -0.020^**^ (0.004) | -0.021^**^ (0.004) | -0.019^**^ (0.004) | -0.012^**^ (0.004) | -0.008^**^ (0.004) | -0.020^**^ (0.004) | -0.012^**^ (0.004) | -0.002 (0.004) | -0.020^**^ (0.004) |
|  |  |  |  |  |  |  |  |  |  |
| Income situation | -0.965^**^ (0.080) | -1.121^**^ (0.080) | -1.316^**^ (0.087) | -0.855^**^ (0.071) | -0.991^**^ (0.080) | -1.102^**^ (0.080) | -0.845^**^ (0.076) | -0.847^**^ (0.072) | -1.102^**^ (0.080) |
| / |  |  |  |  |  |  |  |  |  |
| cut1 | -4.377^**^ (0.763) | -4.512^**^ (0.794) | -4.973^**^ (0.807) | -3.655^**^ (0.661) | -4.071^**^ (0.698) | -6.976^**^ (0.802) | -5.619^**^ (0.729) | -4.080^**^ (0.696) | -6.976^**^ (0.802) |
|  |  |  |  |  |  |  |  |  |  |
| cut2 | -2.496^**^ (0.749) | -2.356^**^ (0.785) | -2.488^**^ (0.790) | -1.938^**^ (0.657) | -2.210^**^ (0.689) | -4.424^**^ (0.779) | -3.838^**^ (0.707) | -1.982^**^ (0.682) | -4.424^**^ (0.779) |
|  |  |  |  |  |  |  |  |  |  |
| cut3 | -0.733 (0.737) | -0.560 (0.781) | -0.577 (0.785) | -0.652 (0.654) | -0.890 (0.688) | -2.410^**^ (0.769) | -1.577^**^ (0.696) | -0.010 (0.676) | -2.410^**^ (0.769) |
| Pseudo *R*^2^ | 0.108 | 0.144 | 0.175 | 0.091 | 0.102 | 0.144 | 0.090 | 0.105 | 0.144 |
| Observations | 1003 | 989 | 990 | 1007 | 1022 | 977 | 993 | 981 | 977 |

*Note*: Ordered logistic modeling coefficients with robust standard errors in parentheses. Reference category for education is “primary & lower secondary”.^*^ *p* < 0.10, ^**^ *p* < 0.05

**AT5b**: Big Five of Personality and Perceived Financial Threat from Covid-19 in Switzerland, Spain and the United Kingdom

|  | **Switzerland** | | | **Spain** | | | **United Kingdom** | | |
| --- | --- | --- | --- | --- | --- | --- | --- | --- | --- |
| Pandemic phase | Spring 2020 | Winter 2020/2021 | Spring 2021 | Spring 2020 | Winter 2020/2021 | Spring 2021 | Spring 2020 | Winter 2020/2021 | Spring 2021 |
|  |  |  |  |  |  |  |  |  |  |
| Openness to experience | -0.162 (0.336) | 0.544^*^ (0.295) | -0.009 (0.323) | -0.230 (0.285) | -0.257 (0.299) | -0.161 (0.319) | 1.082^**^ (0.387) | 0.717^**^ (0.275) | 0.236 (0.299) |
|  |  |  |  |  |  |  |  |  |  |
| Extraversion | 0.213 (0.220) | 0.171 (0.200) | 0.192 (0.224) | 0.091 (0.220) | 0.087 (0.237) | 0.338 (0.211) | -0.068 (0.290) | 0.215 (0.182) | 0.379^**^ (0.189) |
|  |  |  |  |  |  |  |  |  |  |
| Conscientiousness | 0.265 (0.317) | 0.477 (0.320) | -0.127 (0.322) | 0.538^*^ (0.326) | 1.141^**^ (0.338) | 1.004^**^ (0.337) | 0.676^*^ (0.393) | -0.642^**^ (0.258) | -0.209 (0.306) |
|  |  |  |  |  |  |  |  |  |  |
| Agreeableness | 0.040 (0.326) | -0.076 (0.318) | 0.461 (0.354) | 0.359 (0.350) | 0.225 (0.344) | -0.152 (0.353) | -0.524 (0.381) | 0.075 (0.282) | 0.071 (0.304) |
|  |  |  |  |  |  |  |  |  |  |
| Neuroticism | 0.880^**^ (0.194) | 0.562^**^ (0.180) | 0.907^**^ (0.214) | 0.582^**^ (0.200) | 0.605^**^ (0.193) | 0.490^**^ (0.211) | 0.805^**^ (0.274) | 0.523^**^ (0.187) | 0.812^**^ (0.173) |
|  |  |  |  |  |  |  |  |  |  |
| Education |  |  |  |  |  |  |  |  |  |
|  |  |  |  |  |  |  |  |  |  |
| Upper, post secondary | 0.265 (0.237) | 0.674^**^ (0.210) | 0.526^**^ (0.236) | 0.137 (0.165) | -0.118 (0.164) | 0.120 (0.159) | 0.081 (0.165) | 0.515^**^ (0.174) | 0.136 (0.177) |
|  |  |  |  |  |  |  |  |  |  |
| Tertiary | 0.514^**^ (0.247) | 0.819^**^ (0.206) | 0.520^**^ (0.241) | 0.570^**^ (0.149) | -0.133 (0.147) | -0.085 (0.158) | 0.366^**^ (0.144) | 0.615^**^ (0.180) | 0.462^**^ (0.160) |
|  |  |  |  |  |  |  |  |  |  |
| Male | 0.172 (0.127) | -0.082 (0.117) | -0.030 (0.131) | -0.396^**^ (0.122) | -0.280^**^ (0.122) | -0.464^**^ (0.125) | -0.001 (0.125) | -0.128 (0.134) | 0.357^**^ (0.142) |
|  |  |  |  |  |  |  |  |  |  |
| Age | -0.017^**^ (0.004) | -0.021^**^ (0.004) | -0.022^**^ (0.004) | -0.013^**^ (0.004) | -0.020^**^ (0.004) | -0.024^**^ (0.004) | -0.021^**^ (0.004) | -0.017^**^ (0.005) | -0.029^**^ (0.004) |
|  |  |  |  |  |  |  |  |  |  |
| Income situation | -0.894^**^ (0.077) | -0.745^**^ (0.067) | -1.013^**^ (0.079) | -0.933^**^ (0.072) | -0.970^**^ (0.075) | -1.242^**^ (0.079) | -1.061^**^ (0.073) | -1.210^**^ (0.073) | -0.980^**^ (0.078) |
| / |  |  |  |  |  |  |  |  |  |
| cut1 | -3.323^**^ (0.792) | -1.895^**^ (0.724) | -3.491^**^ (0.802) | -4.624^**^ (0.816) | -4.598^**^ (0.881) | -6.117^**^ (0.879) | -3.349^**^ (0.910) | -4.302^**^ (0.691) | -3.566^**^ (0.725) |
|  |  |  |  |  |  |  |  |  |  |
| cut2 | -1.428^*^ (0.784) | 0.146 (0.723) | -1.223 (0.795) | -2.821^**^ (0.800) | -2.389^**^ (0.861) | -3.348^**^ (0.856) | -1.397 (0.904) | -2.394^**^ (0.682) | -1.564^**^ (0.714) |
|  |  |  |  |  |  |  |  |  |  |
| cut3 | 0.187 (0.782) | 1.725^**^ (0.723) | 0.589 (0.785) | -0.745 (0.792) | -0.245 (0.854) | -1.091 (0.848) | -0.260 (0.904) | -1.027 (0.675) | -0.567 (0.709) |
| Pseudo *R*^2^ | 0.106 | 0.091 | 0.145 | 0.112 | 0.128 | 0.174 | 0.155 | 0.190 | 0.164 |
| Observations | 990 | 1120 | 990 | 992 | 1002 | 1016 | 1000 | 1029 | 986 |

*Note*: Ordered logistic modeling coefficients with robust standard errors in parentheses. Reference category for education is “primary & lower secondary”. ^*^ *p* < 0.10, ^**^ *p* < 0.05

**AF3**: Descriptive Overview: Perception of Covid-19 as a (very) major social threat


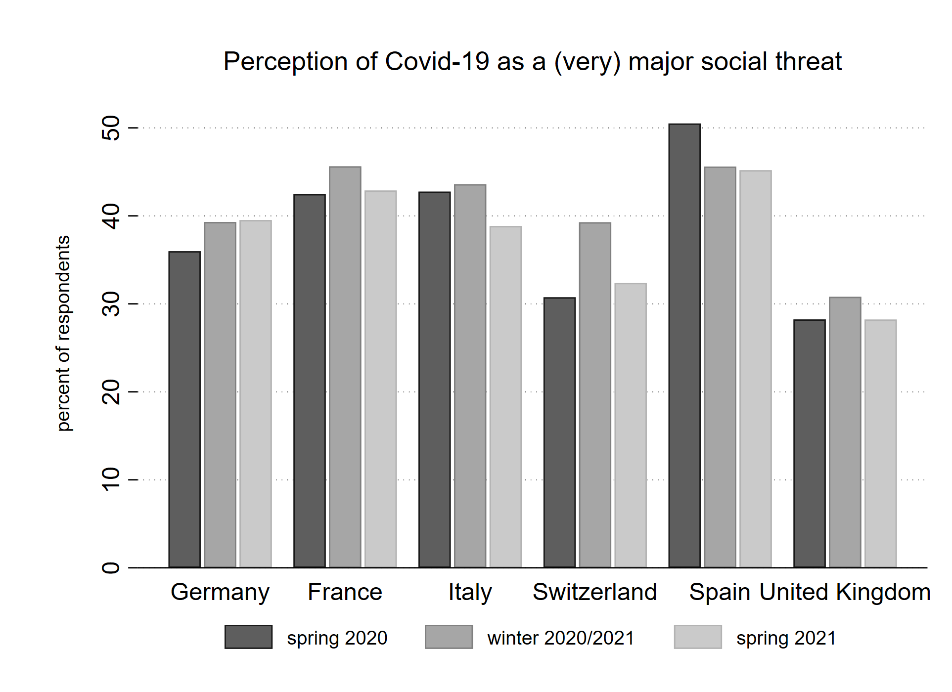


*Note*: The proportions displayed equal the share of respondents per country and

pandemic phase who perceive Covid-19 as a (very) major social threat.

**AT6a**: Big Five of Personality and Perceived Social Threat from Covid-19 in Germany, France and Italy

|  | **Germany** | | | **France** | | | **Italy** | | |
| --- | --- | --- | --- | --- | --- | --- | --- | --- | --- |
| Pandemic phase | Spring 2020 | Winter 2020/2021 | Spring 2021 | Spring 2020 | Winter 2020/2021 | Spring 2021 | Spring 2020 | Winter 2020/2021 | Spring 2021 |
|  |  |  |  |  |  |  |  |  |  |
| Openness to experience | 0.316 (0.310) | 0.176 (0.322) | 0.112 (0.293) | -0.499^*^ (0.279) | 0.535^**^ (0.265) | -0.089 (0.313) | -0.435^*^ (0.265) | -0.194 (0.277) | -0.089 (0.313) |
|  |  |  |  |  |  |  |  |  |  |
| Extraversion | 0.526^**^ (0.199) | -0.017 (0.217) | 0.113 (0.198) | 0.692^**^ (0.204) | 0.322 (0.199) | 0.191 (0.209) | 0.338^*^ (0.176) | 0.215 (0.199) | 0.191 (0.209) |
|  |  |  |  |  |  |  |  |  |  |
| Conscientiousness | -0.414 (0.311) | 0.213 (0.323) | 0.151 (0.352) | 0.006 (0.303) | 0.632^**^ (0.298) | -0.699^**^ (0.332) | -0.101 (0.309) | -0.080 (0.299) | -0.699^**^ (0.332) |
|  |  |  |  |  |  |  |  |  |  |
| Agreeableness | 0.292 (0.365) | -0.121 (0.343) | -0.170 (0.386) | -0.163 (0.354) | -0.134 (0.364) | -0.102 (0.328) | -0.236 (0.301) | 0.291 (0.311) | -0.102 (0.328) |
|  |  |  |  |  |  |  |  |  |  |
| Neuroticism | 0.575^**^ (0.179) | 0.497^**^ (0.197) | 0.522^**^ (0.193) | 0.687^**^ (0.192) | 0.870^**^ (0.204) | 0.983^**^ (0.232) | 0.479^**^ (0.187) | 1.212^**^ (0.200) | 0.983^**^ (0.232) |
|  |  |  |  |  |  |  |  |  |  |
| Education |  |  |  |  |  |  |  |  |  |
|  |  |  |  |  |  |  |  |  |  |
| Upper, post secondary | -0.112 (0.206) | 0.002 (0.197) | -0.162 (0.168) | -0.045 (0.171) | 0.152 (0.164) | 0.295^*^ (0.151) | -0.020 (0.129) | 0.051 (0.138) | 0.295^*^ (0.151) |
|  |  |  |  |  |  |  |  |  |  |
| Tertiary | 0.219 (0.220) | 0.044 (0.203) | -0.044 (0.183) | 0.035 (0.189) | 0.277 (0.172) | 0.511^**^ (0.178) | -0.001 (0.186) | 0.592^**^ (0.161) | 0.511^**^ (0.178) |
|  |  |  |  |  |  |  |  |  |  |
| Male | 0.020 (0.128) | -0.275^**^ (0.133) | -0.057 (0.127) | 0.088 (0.124) | -0.233^*^ (0.123) | 0.025 (0.124) | -0.177 (0.122) | -0.202^*^ (0.119) | 0.025 (0.124) |
|  |  |  |  |  |  |  |  |  |  |
| Age | -0.016^**^ (0.004) | -0.012^**^ (0.004) | -0.021^**^ (0.004) | -0.017^**^ (0.004) | 0.000 (0.004) | -0.025^**^ (0.004) | -0.011^**^ (0.004) | -0.007^**^ (0.004) | -0.025^**^ (0.004) |
|  |  |  |  |  |  |  |  |  |  |
| Income situation | -0.320^**^ (0.063) | -0.275^**^ (0.070) | -0.279^**^ (0.066) | -0.344^**^ (0.068) | -0.220^**^ (0.066) | -0.277^**^ (0.069) | -0.217^**^ (0.065) | -0.210^**^ (0.069) | -0.277^**^ (0.069) |
| / |  |  |  |  |  |  |  |  |  |
| cut1 | -1.825^**^ (0.717) | -2.312^**^ (0.805) | -2.910^**^ (0.776) | -2.701^**^ (0.708) | 0.192 (0.719) | -3.540^**^ (0.773) | -2.743^**^ (0.644) | -1.141 (0.696) | -3.540^**^ (0.773) |
|  |  |  |  |  |  |  |  |  |  |
| cut2 | 0.056 (0.713) | -0.380 (0.799) | -0.941 (0.770) | -0.945 (0.702) | 2.018^**^ (0.725) | -1.307^*^ (0.771) | -1.085^*^ (0.640) | 0.778 (0.693) | -1.307^*^ (0.771) |
|  |  |  |  |  |  |  |  |  |  |
| cut3 | 1.837^**^ (0.715) | 1.576^**^ (0.794) | 0.830 (0.770) | 0.799 (0.698) | 4.170^**^ (0.741) | 0.720 (0.773) | 0.621 (0.644) | 2.632^**^ (0.695) | 0.720 (0.773) |
| Pseudo *R*^2^ | 0.035 | 0.027 | 0.033 | 0.039 | 0.024 | 0.063 | 0.022 | 0.038 | 0.063 |
| Observations | 1003 | 989 | 990 | 1007 | 1022 | 977 | 993 | 981 | 977 |

*Note*: Ordered logistic modeling coefficients with robust standard errors in parentheses. Reference category for education is “primary & lower secondary”. ^*^ *p* < 0.10, ^**^ *p* < 0.05

**AT6b**: Big Five of Personality and Perceived Social Threat from Covid-19 in Switzerland, Spain and the United Kingdom

|  | **Switzerland** | | | **Spain** | | | **United Kingdom** | | |
| --- | --- | --- | --- | --- | --- | --- | --- | --- | --- |
| Pandemic phase | Spring 2020 | Winter 2020/2021 | Spring 2021 | Spring 2020 | Winter 2020/2021 | Spring 2021 | Spring 2020 | Winter 2020/2021 | Spring 2021 |
|  |  |  |  |  |  |  |  |  |  |
| Openness to experience | -0.476 (0.346) | -0.192 (0.264) | -0.231 (0.324) | -0.433 (0.276) | -1.050^**^ (0.306) | -0.656^**^ (0.253) | 0.196 (0.433) | -0.091 (0.268) | 0.399 (0.279) |
|  |  |  |  |  |  |  |  |  |  |
| Extraversion | 0.495^**^ (0.207) | 0.612^**^ (0.189) | 0.335 (0.239) | 0.344 (0.221) | 0.429^*^ (0.232) | 0.039 (0.208) | 0.805^**^ (0.278) | 0.497^**^ (0.182) | 0.331^*^ (0.184) |
|  |  |  |  |  |  |  |  |  |  |
| Conscientiousness | -0.221 (0.342) | 0.372 (0.298) | -0.207 (0.307) | -0.087 (0.306) | 0.573^*^ (0.323) | 0.163 (0.298) | -0.275 (0.395) | 0.026 (0.293) | -0.614^**^ (0.298) |
|  |  |  |  |  |  |  |  |  |  |
| Agreeableness | -0.017 (0.331) | 0.363 (0.294) | -0.081 (0.364) | -0.806^**^ (0.329) | -0.225 (0.402) | -0.318 (0.368) | -0.188 (0.411) | -0.045 (0.312) | 0.002 (0.315) |
|  |  |  |  |  |  |  |  |  |  |
| Neuroticism | 0.910^**^ (0.193) | 0.683^**^ (0.185) | 0.787^**^ (0.208) | 0.481^**^ (0.200) | 0.763^**^ (0.210) | 0.694^**^ (0.206) | 1.579^**^ (0.267) | 0.581^**^ (0.186) | 0.868^**^ (0.181) |
|  |  |  |  |  |  |  |  |  |  |
| Education |  |  |  |  |  |  |  |  |  |
|  |  |  |  |  |  |  |  |  |  |
| Upper, post secondary | -0.110 (0.229) | -0.138 (0.201) | 0.477^*^ (0.249) | 0.096 (0.164) | 0.033 (0.165) | 0.404^**^ (0.168) | -0.143 (0.170) | 0.336^**^ (0.165) | 0.112 (0.177) |
|  |  |  |  |  |  |  |  |  |  |
| Tertiary | 0.222 (0.236) | 0.043 (0.206) | 0.683^**^ (0.253) | 0.100 (0.142) | 0.152 (0.149) | 0.296^*^ (0.161) | 0.146 (0.145) | 0.379^**^ (0.156) | 0.390^**^ (0.162) |
|  |  |  |  |  |  |  |  |  |  |
| Male | 0.167 (0.128) | -0.091 (0.117) | -0.001 (0.128) | -0.221^*^ (0.117) | -0.272^**^ (0.119) | -0.067 (0.121) | -0.001 (0.125) | -0.154 (0.126) | 0.229^*^ (0.135) |
|  |  |  |  |  |  |  |  |  |  |
| Age | -0.008^**^ (0.004) | -0.004 (0.003) | -0.012^**^ (0.004) | -0.009^**^ (0.004) | -0.010^**^ (0.004) | -0.008^*^ (0.004) | -0.015^**^ (0.004) | -0.018^**^ (0.004) | -0.032^**^ (0.004) |
|  |  |  |  |  |  |  |  |  |  |
| Income situation | -0.250^**^ (0.065) | -0.300^**^ (0.062) | -0.248^**^ (0.065) | -0.123^*^ (0.066) | -0.148^**^ (0.061) | -0.215^**^ (0.063) | -0.418^**^ (0.067) | -0.308^**^ (0.056) | -0.291^**^ (0.065) |
| / |  |  |  |  |  |  |  |  |  |
| cut1 | -1.813^**^ (0.774) | -0.786 (0.684) | -1.839^**^ (0.781) | -3.317^**^ (0.728) | -2.480^**^ (0.862) | -3.048^**^ (0.740) | -0.537 (0.921) | -1.434^**^ (0.689) | -1.844^**^ (0.711) |
|  |  |  |  |  |  |  |  |  |  |
| cut2 | 0.130 (0.772) | 1.167^*^ (0.689) | 0.241 (0.781) | -1.675^**^ (0.725) | -0.433 (0.858) | -0.908 (0.735) | 1.041 (0.918) | 0.138 (0.688) | -0.344 (0.709) |
|  |  |  |  |  |  |  |  |  |  |
| cut3 | 2.011^**^ (0.781) | 3.061^**^ (0.700) | 2.211^**^ (0.775) | -0.025 (0.723) | 1.405 (0.859) | 1.205 (0.740) | 2.349^**^ (0.917) | 1.541^**^ (0.690) | 1.225^*^ (0.709) |
| Pseudo *R*^2^ | 0.032 | 0.024 | 0.035 | 0.019 | 0.029 | 0.023 | 0.069 | 0.054 | 0.089 |
| Observations | 990 | 1120 | 990 | 992 | 1002 | 1016 | 1000 | 1029 | 986 |

*Note*: Ordered logistic modeling coefficients with robust standard errors in parentheses. Reference category for education is “primary & lower secondary”. ^*^ *p* < 0.10, ^**^ *p* < 0.05

**AF4**: Descriptive Overview: Average levels of experienced fear


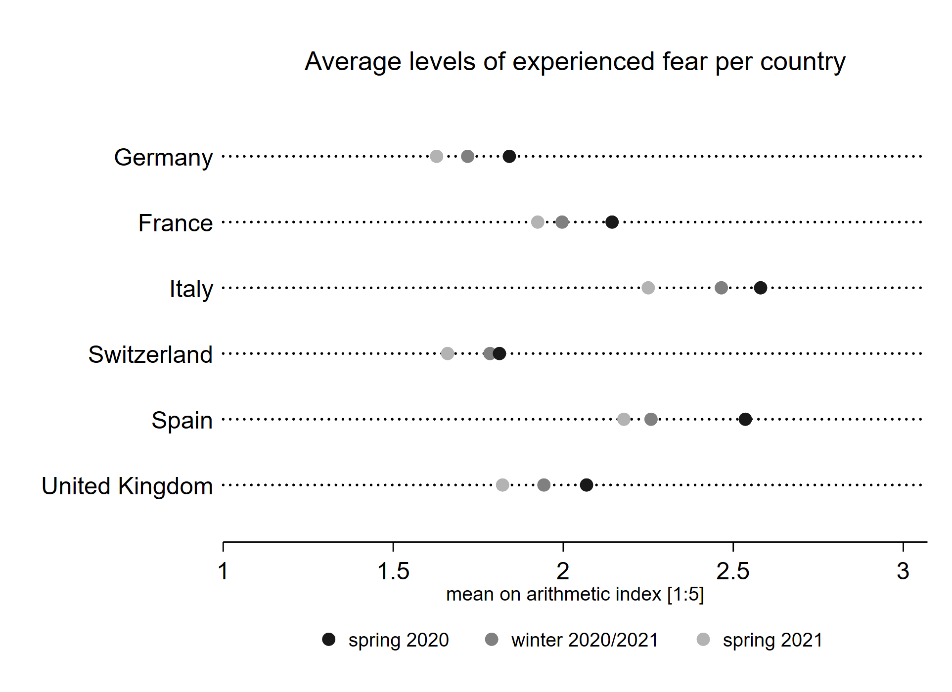


*Note*: Displayed are the mean levels of experienced fear per country and pandemic phase.

**AT7a**: Big Five of Personality and Fear in Germany, France and Italy

|  | **Germany** | | | **France** | | | **Italy** | | |
| --- | --- | --- | --- | --- | --- | --- | --- | --- | --- |
| Pandemic phase | Spring 2020 | Winter 2020/2021 | Spring 2021 | Spring 2020 | Winter 2020/2021 | Spring 2021 | Spring 2020 | Winter 2020/2021 | Spring 2021 |
|  |  |  |  |  |  |  |  |  |  |
| Openness to experience | 0.176 (0.134) | -0.212 (0.137) | -0.016 (0.122) | 0.049 (0.120) | 0.030 (0.124) | -0.040 (0.133) | -0.141 (0.137) | -0.122 (0.139) | 0.009 (0.125) |
|  |  |  |  |  |  |  |  |  |  |
| Extraversion | 0.048 (0.086) | -0.023 (0.086) | 0.027 (0.088) | 0.006 (0.095) | -0.258^**^ (0.085) | -0.212^**^ (0.086) | -0.238^**^ (0.092) | -0.260^**^ (0.101) | -0.289^**^ (0.091) |
|  |  |  |  |  |  |  |  |  |  |
| Conscientiousness | -0.099 (0.142) | 0.102 (0.145) | 0.078 (0.151) | 0.057 (0.135) | 0.141 (0.176) | -0.012 (0.170) | 0.096 (0.163) | -0.215 (0.185) | 0.029 (0.148) |
|  |  |  |  |  |  |  |  |  |  |
| Agreeableness | 0.083 (0.150) | 0.214 (0.149) | 0.156 (0.156) | -0.164 (0.157) | -0.356^**^ (0.162) | -0.367^**^ (0.168) | -0.069 (0.148) | 0.110 (0.155) | -0.000 (0.150) |
|  |  |  |  |  |  |  |  |  |  |
| Neuroticism | 0.835^**^ (0.086) | 0.833^**^ (0.078) | 0.795^**^ (0.074) | 0.971^**^ (0.078) | 0.948^**^ (0.084) | 0.992^**^ (0.080) | 0.848^**^ (0.094) | 0.881^**^ (0.106) | 0.976^**^ (0.082) |
|  |  |  |  |  |  |  |  |  |  |
| Education |  |  |  |  |  |  |  |  |  |
|  |  |  |  |  |  |  |  |  |  |
| Upper, post secondary | -0.074 (0.090) | 0.141^**^ (0.071) | -0.041 (0.074) | 0.125 (0.077) | 0.162^**^ (0.071) | 0.045 (0.070) | 0.002 (0.068) | 0.147^**^ (0.073) | 0.076 (0.074) |
|  |  |  |  |  |  |  |  |  |  |
| Tertiary | 0.100 (0.095) | 0.216^**^ (0.076) | -0.027 (0.080) | 0.150^*^ (0.088) | 0.184^**^ (0.080) | 0.045 (0.081) | 0.156^*^ (0.085) | 0.397^**^ (0.093) | 0.112 (0.094) |
|  |  |  |  |  |  |  |  |  |  |
| Male | -0.038 (0.058) | -0.125^**^ (0.055) | -0.105^*^ (0.055) | -0.069 (0.059) | -0.092^*^ (0.053) | -0.065 (0.054) | -0.258^**^ (0.063) | -0.222^**^ (0.066) | -0.131^**^ (0.062) |
|  |  |  |  |  |  |  |  |  |  |
| Age | -0.007^**^ (0.002) | -0.006^**^ (0.002) | -0.006^**^ (0.002) | -0.007^**^ (0.002) | -0.007^**^ (0.002) | -0.005^**^ (0.002) | -0.002 (0.002) | -0.004^**^ (0.002) | -0.009^**^ (0.002) |
|  |  |  |  |  |  |  |  |  |  |
| Income situation | -0.162^**^ (0.027) | -0.121^**^ (0.025) | -0.147^**^ (0.027) | -0.102^**^ (0.031) | -0.088^**^ (0.028) | -0.131^**^ (0.029) | -0.090^**^ (0.032) | -0.128^**^ (0.034) | -0.100^**^ (0.031) |
|  |  |  |  |  |  |  |  |  |  |
| Constant | 1.804^**^ (0.335) | 1.546^**^ (0.384) | 1.516^**^ (0.330) | 1.897^**^ (0.328) | 2.075^**^ (0.353) | 2.346^**^ (0.322) | 2.608^**^ (0.340) | 2.713^**^ (0.421) | 2.328^**^ (0.331) |
| *R*^2^ | 0.218 | 0.254 | 0.209 | 0.195 | 0.217 | 0.237 | 0.169 | 0.182 | 0.201 |
| Adjusted *R*^2^ | 0.210 | 0.246 | 0.201 | 0.186 | 0.209 | 0.229 | 0.160 | 0.173 | 0.193 |
| Observations | 1003 | 989 | 990 | 1007 | 1022 | 983 | 993 | 981 | 977 |

*Note*: Linear regression coefficients with robust standard errors in parentheses. Reference category for education is “primary & lower secondary”. ^*^ *p* < 0.10, ^**^ *p* < 0.05

**AT7b**: Big Five of Personality and Fear in Switzerland, Spain and the United Kingdom

|  | **Switzerland** | | | **Spain** | | | **United Kingdom** | | |
| --- | --- | --- | --- | --- | --- | --- | --- | --- | --- |
| Pandemic phase | Spring 2020 | Winter 2020/2021 | Spring 2021 | Spring 2020 | Winter 2020/2021 | Spring 2021 | Spring 2020 | Winter 2020/2021 | Spring 2021 |
|  |  |  |  |  |  |  |  |  |  |
| Openness to experience | -0.214 (0.134) | -0.207^*^ (0.112) | -0.065 (0.114) | -0.037 (0.137) | -0.129 (0.127) | -0.215^*^ (0.120) | -0.413^**^ (0.188) | -0.163 (0.132) | -0.197^*^ (0.119) |
|  |  |  |  |  |  |  |  |  |  |
| Extraversion | -0.078 (0.079) | 0.101 (0.069) | -0.070 (0.079) | 0.122 (0.108) | -0.013 (0.107) | -0.028 (0.091) | 0.069 (0.128) | -0.085 (0.085) | 0.011 (0.086) |
|  |  |  |  |  |  |  |  |  |  |
| Conscientiousness | -0.076 (0.140) | -0.188 (0.126) | 0.046 (0.131) | 0.398^**^ (0.155) | 0.490^**^ (0.143) | 0.268^*^ (0.146) | 0.104 (0.202) | -0.240 (0.161) | -0.460^**^ (0.143) |
|  |  |  |  |  |  |  |  |  |  |
| Agreeableness | 0.196 (0.133) | 0.065 (0.125) | 0.200 (0.129) | -0.304^*^ (0.169) | -0.225 (0.159) | 0.138 (0.187) | 0.266 (0.203) | 0.181 (0.143) | 0.212 (0.137) |
|  |  |  |  |  |  |  |  |  |  |
| Neuroticism | 0.926^**^ (0.077) | 0.761^**^ (0.073) | 0.792^**^ (0.074) | 1.062^**^ (0.095) | 1.032^**^ (0.090) | 0.954^**^ (0.095) | 1.300^**^ (0.129) | 0.763^**^ (0.074) | 0.765^**^ (0.081) |
|  |  |  |  |  |  |  |  |  |  |
| Education |  |  |  |  |  |  |  |  |  |
|  |  |  |  |  |  |  |  |  |  |
| Upper, post secondary | -0.049 (0.089) | 0.047 (0.085) | -0.091 (0.105) | 0.123 (0.083) | 0.087 (0.076) | 0.036 (0.078) | -0.039 (0.083) | 0.139^*^ (0.081) | -0.048 (0.081) |
|  |  |  |  |  |  |  |  |  |  |
| Tertiary | 0.133 (0.092) | 0.144^*^ (0.084) | -0.159 (0.105) | 0.155^**^ (0.070) | 0.079 (0.073) | 0.156^**^ (0.073) | 0.058 (0.071) | 0.124^*^ (0.074) | 0.015 (0.073) |
|  |  |  |  |  |  |  |  |  |  |
| Male | -0.043 (0.050) | 0.006 (0.046) | 0.035 (0.049) | -0.312^**^ (0.060) | -0.236^**^ (0.059) | -0.226^**^ (0.060) | 0.000 (0.063) | -0.190^**^ (0.064) | 0.151^**^ (0.064) |
|  |  |  |  |  |  |  |  |  |  |
| Age | -0.008^**^ (0.001) | -0.010^**^ (0.001) | -0.009^**^ (0.001) | -0.011^**^ (0.002) | -0.008^**^ (0.002) | -0.009^**^ (0.002) | -0.008^**^ (0.002) | -0.009^**^ (0.002) | -0.013^**^ (0.002) |
|  |  |  |  |  |  |  |  |  |  |
| Income situation | -0.096^**^ (0.024) | -0.120^**^ (0.023) | -0.099^**^ (0.024) | -0.057^**^ (0.029) | -0.161^**^ (0.029) | -0.152^**^ (0.030) | -0.140^**^ (0.031) | -0.124^**^ (0.028) | -0.120^**^ (0.032) |
|  |  |  |  |  |  |  |  |  |  |
| Constant | 1.948^**^ (0.329) | 2.255^**^ (0.291) | 1.709^**^ (0.334) | 2.088^**^ (0.399) | 2.062^**^ (0.374) | 2.058^**^ (0.389) | 1.584^**^ (0.466) | 2.434^**^ (0.317) | 2.706^**^ (0.327) |
| *R*^2^ | 0.266 | 0.241 | 0.235 | 0.226 | 0.235 | 0.207 | 0.196 | 0.232 | 0.245 |
| Adjusted *R*^2^ | 0.259 | 0.234 | 0.227 | 0.218 | 0.228 | 0.199 | 0.187 | 0.225 | 0.237 |
| Observations | 990 | 1120 | 990 | 992 | 1002 | 1016 | 1000 | 1029 | 986 |

*Note*: Linear regression coefficients with robust standard errors in parentheses. Reference category for education is “primary & lower secondary”. ^*^ *p* < 0.10, ^**^ *p* < 0.05

**AF5**: Descriptive Overview: Average levels of experienced anger


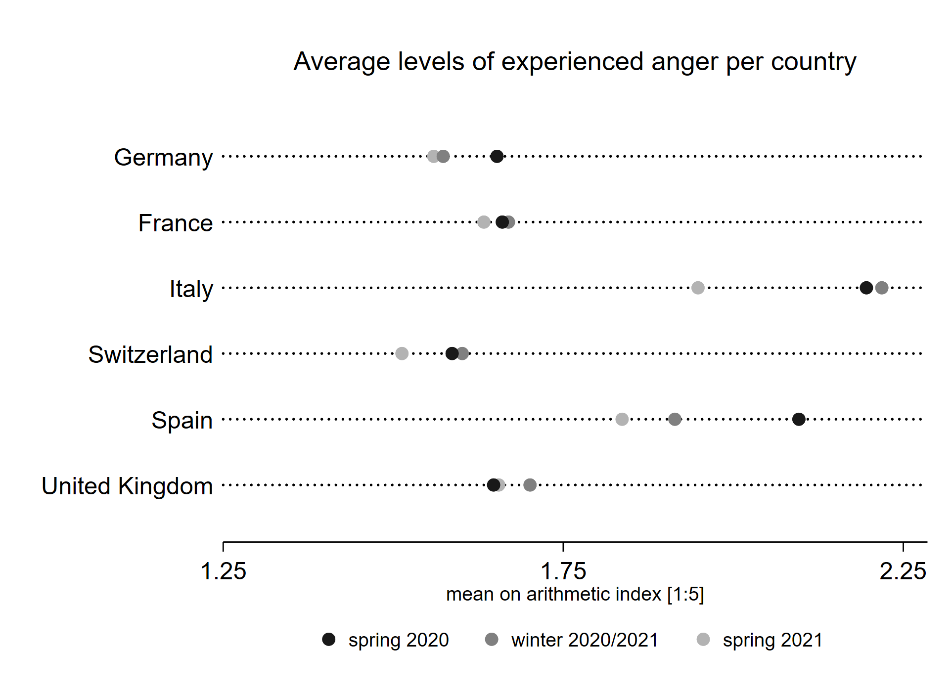


*Note*: Displayed are the mean levels of experienced anger per country and pandemic phase.

**AT8a**: Big Five of Personality and Anger in Germany, France and Italy

|  | **Germany** | | | **France** | | | **Italy** | | |
| --- | --- | --- | --- | --- | --- | --- | --- | --- | --- |
| Pandemic phase | Spring 2020 | Winter 2020/2021 | Spring 2021 | Spring 2020 | Winter 2020/2021 | Spring 2021 | Spring 2020 | Winter 2020/2021 | Spring 2021 |
|  |  |  |  |  |  |  |  |  |  |
| Openness to experience | -0.267^*^ (0.145) | -0.241^*^ (0.129) | -0.106 (0.123) | 0.014 (0.119) | -0.026 (0.114) | -0.102 (0.123) | -0.107 (0.142) | -0.179 (0.150) | 0.060 (0.120) |
|  |  |  |  |  |  |  |  |  |  |
| Extraversion | 0.140 (0.092) | -0.018 (0.089) | 0.101 (0.081) | 0.365^**^ (0.084) | 0.031 (0.080) | -0.082 (0.077) | -0.123 (0.102) | -0.145 (0.106) | -0.101 (0.099) |
|  |  |  |  |  |  |  |  |  |  |
| Conscientiousness | 0.023 (0.140) | 0.002 (0.149) | 0.128 (0.147) | 0.029 (0.133) | -0.036 (0.140) | -0.039 (0.152) | 0.227 (0.171) | 0.297 (0.187) | 0.140 (0.147) |
|  |  |  |  |  |  |  |  |  |  |
| Agreeableness | -0.552^**^ (0.166) | -0.479^**^ (0.164) | -0.736^**^ (0.157) | -0.515^**^ (0.143) | -0.686^**^ (0.152) | -0.803^**^ (0.152) | -0.571^**^ (0.164) | -0.959^**^ (0.177) | -0.939^**^ (0.160) |
|  |  |  |  |  |  |  |  |  |  |
| Neuroticism | 0.326^**^ (0.086) | 0.300^**^ (0.073) | 0.361^**^ (0.077) | 0.503^**^ (0.070) | 0.360^**^ (0.075) | 0.571^**^ (0.078) | 0.528^**^ (0.096) | 0.600^**^ (0.111) | 0.592^**^ (0.083) |
|  |  |  |  |  |  |  |  |  |  |
| Education |  |  |  |  |  |  |  |  |  |
|  |  |  |  |  |  |  |  |  |  |
| Upper, post secondary | -0.092 (0.080) | 0.080 (0.071) | -0.096 (0.079) | 0.009 (0.076) | 0.169^**^ (0.070) | -0.035 (0.071) | -0.030 (0.070) | 0.088 (0.076) | 0.106 (0.070) |
|  |  |  |  |  |  |  |  |  |  |
| Tertiary | 0.099 (0.089) | 0.134^*^ (0.076) | -0.159^*^ (0.083) | -0.065 (0.088) | 0.163^**^ (0.079) | -0.047 (0.082) | -0.023 (0.091) | 0.400^**^ (0.095) | 0.136 (0.086) |
|  |  |  |  |  |  |  |  |  |  |
| Male | 0.106^*^ (0.058) | -0.013 (0.052) | -0.006 (0.055) | -0.046 (0.057) | 0.023 (0.056) | 0.090^*^ (0.053) | -0.126^**^ (0.064) | -0.082 (0.069) | -0.047 (0.061) |
|  |  |  |  |  |  |  |  |  |  |
| Age | -0.006^**^ (0.002) | -0.007^**^ (0.001) | -0.007^**^ (0.002) | -0.006^**^ (0.002) | -0.003 (0.002) | -0.001 (0.002) | -0.005^**^ (0.002) | -0.004^*^ (0.002) | -0.009^**^ (0.002) |
|  |  |  |  |  |  |  |  |  |  |
| Income situation | -0.133^**^ (0.029) | -0.136^**^ (0.025) | -0.139^**^ (0.026) | -0.071^**^ (0.032) | -0.108^**^ (0.029) | -0.097^**^ (0.028) | -0.109^**^ (0.035) | -0.157^**^ (0.035) | -0.129^**^ (0.030) |
|  |  |  |  |  |  |  |  |  |  |
| Constant | 2.947^**^ (0.361) | 2.989^**^ (0.371) | 2.973^**^ (0.363) | 1.962^**^ (0.308) | 2.551^**^ (0.305) | 2.698^**^ (0.314) | 3.008^**^ (0.347) | 3.381^**^ (0.429) | 3.207^**^ (0.324) |
| *R*^2^ | 0.138 | 0.148 | 0.151 | 0.123 | 0.101 | 0.164 | 0.116 | 0.155 | 0.201 |
| Adjusted *R*^2^ | 0.130 | 0.140 | 0.142 | 0.114 | 0.092 | 0.155 | 0.107 | 0.147 | 0.193 |
| Observations | 1003 | 989 | 990 | 1007 | 1022 | 983 | 993 | 981 | 977 |

*Note*: Linear regression coefficients with robust standard errors in parentheses. Reference category for education is “primary & lower secondary”. ^*^ *p* < 0.10, ^**^ *p* < 0.05

**AT8b**: Big Five of Personality and Anger in Switzerland, Spain and the United Kingdom

|  | **Switzerland** | | | **Spain** | | | **United Kingdom** | | |
| --- | --- | --- | --- | --- | --- | --- | --- | --- | --- |
| Pandemic phase | Spring 2020 | Winter 2020/2021 | Spring 2021 | Spring 2020 | Winter 2020/2021 | Spring 2021 | Spring 2020 | Winter 2020/2021 | Spring 2021 |
|  |  |  |  |  |  |  |  |  |  |
| Openness to experience | 0.181 (0.140) | -0.144 (0.107) | -0.073 (0.106) | -0.320^**^ (0.134) | -0.237^*^ (0.126) | -0.182 (0.121) | -0.190 (0.171) | -0.054 (0.116) | -0.178 (0.112) |
|  |  |  |  |  |  |  |  |  |  |
| Extraversion | -0.035 (0.082) | 0.091 (0.068) | 0.098 (0.081) | 0.181^*^ (0.110) | -0.026 (0.111) | -0.125 (0.087) | 0.069 (0.100) | 0.013 (0.073) | 0.165^**^ (0.072) |
|  |  |  |  |  |  |  |  |  |  |
| Conscientiousness | -0.106 (0.141) | -0.165 (0.136) | -0.150 (0.141) | 0.004 (0.152) | 0.150 (0.161) | -0.007 (0.130) | -0.159 (0.174) | -0.276^**^ (0.141) | -0.649^**^ (0.138) |
|  |  |  |  |  |  |  |  |  |  |
| Agreeableness | -0.400^**^ (0.138) | -0.713^**^ (0.130) | -0.565^**^ (0.134) | -0.705^**^ (0.158) | -0.256 (0.172) | -0.209 (0.168) | -0.326^*^ (0.184) | -0.372^**^ (0.138) | 0.041 (0.138) |
|  |  |  |  |  |  |  |  |  |  |
| Neuroticism | 0.514^**^ (0.077) | 0.324^**^ (0.064) | 0.280^**^ (0.065) | 0.597^**^ (0.093) | 0.721^**^ (0.082) | 0.450^**^ (0.089) | 0.854^**^ (0.101) | 0.426^**^ (0.066) | 0.548^**^ (0.070) |
|  |  |  |  |  |  |  |  |  |  |
| Education |  |  |  |  |  |  |  |  |  |
|  |  |  |  |  |  |  |  |  |  |
| Upper, post secondary | -0.133 (0.103) | 0.041 (0.073) | -0.078 (0.105) | 0.096 (0.079) | 0.075 (0.075) | 0.021 (0.072) | -0.042 (0.067) | 0.206^**^ (0.073) | -0.000 (0.077) |
|  |  |  |  |  |  |  |  |  |  |
| Tertiary | -0.049 (0.105) | 0.123 (0.076) | -0.099 (0.108) | 0.153^**^ (0.071) | 0.114^*^ (0.066) | 0.077 (0.070) | 0.006 (0.056) | 0.115^*^ (0.066) | -0.012 (0.068) |
|  |  |  |  |  |  |  |  |  |  |
| Male | 0.034 (0.052) | 0.085^*^ (0.047) | 0.128^**^ (0.047) | -0.119^**^ (0.060) | -0.072 (0.058) | -0.051 (0.056) | -0.042 (0.049) | -0.023 (0.058) | 0.208^**^ (0.060) |
|  |  |  |  |  |  |  |  |  |  |
| Age | -0.006^**^ (0.001) | -0.007^**^ (0.001) | -0.007^**^ (0.001) | -0.007^**^ (0.002) | -0.004^**^ (0.002) | -0.005^**^ (0.002) | -0.007^**^ (0.001) | -0.009^**^ (0.002) | -0.013^**^ (0.002) |
|  |  |  |  |  |  |  |  |  |  |
| Income situation | -0.074^**^ (0.026) | -0.074^**^ (0.024) | -0.114^**^ (0.025) | -0.071^**^ (0.028) | -0.138^**^ (0.027) | -0.097^**^ (0.029) | -0.119^**^ (0.026) | -0.107^**^ (0.025) | -0.087^**^ (0.030) |
|  |  |  |  |  |  |  |  |  |  |
| Constant | 2.249^**^ (0.339) | 3.051^**^ (0.277) | 2.949^**^ (0.318) | 3.119^**^ (0.369) | 2.255^**^ (0.366) | 2.558^**^ (0.364) | 2.315^**^ (0.379) | 2.885^**^ (0.281) | 2.836^**^ (0.299) |
| *R*^2^ | 0.128 | 0.158 | 0.158 | 0.138 | 0.142 | 0.079 | 0.202 | 0.196 | 0.234 |
| Adjusted *R*^2^ | 0.119 | 0.150 | 0.150 | 0.130 | 0.133 | 0.070 | 0.194 | 0.188 | 0.226 |
| Observations | 990 | 1120 | 990 | 992 | 1002 | 1016 | 1000 | 1029 | 986 |

*Note*: Linear regression coefficients with robust standard errors in parentheses. Reference category for education is “primary & lower secondary”. ^*^ *p* < 0.10, ^**^ *p* < 0.05

**AF6**: Descriptive Overview: Self-Infection


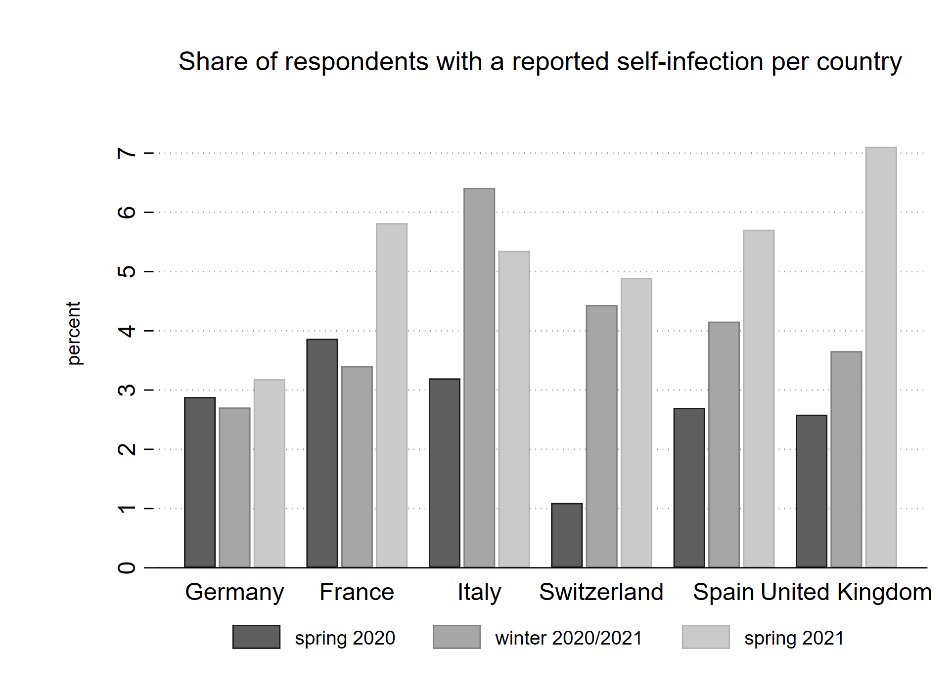


*Note*: The proportions displayed equal the share of respondents per country and

pandemic phase who reported a self-infection with Covid-19.

**AT9a**: Big Five of Personality and Self-Infection with Covid-19 in Germany, France and Italy

|  | **Germany** | | | **France** | | | **Italy** | | |
| --- | --- | --- | --- | --- | --- | --- | --- | --- | --- |
| Pandemic phase | Spring 2020 | Winter 2020/2021 | Spring 2021 | Spring 2020 | Winter 2020/2021 | Spring 2021 | Spring 2020 | Winter 2020/2021 | Spring 2021 |
|  |  |  |  |  |  |  |  |  |  |
| Openness to experience | -0.869 (0.680) | -0.460 (0.903) | -0.110 (0.785) | -1.414^**^ (0.595) | 0.315 (0.752) | 0.098 (0.560) | -0.363 (0.671) | -0.280 (0.494) | 0.008 (0.567) |
|  |  |  |  |  |  |  |  |  |  |
| Extraversion | 0.115 (0.564) | 1.102^*^ (0.604) | 0.971 (0.644) | 0.251 (0.610) | 0.014 (0.458) | 0.515 (0.481) | 1.344^**^ (0.636) | 1.589^**^ (0.397) | 0.530 (0.501) |
|  |  |  |  |  |  |  |  |  |  |
| Conscientiousness | -1.593^**^ (0.540) | -1.399^*^ (0.738) | 0.375 (0.738) | -0.232 (0.661) | -2.789^**^ (0.613) | -0.696 (0.510) | -1.271^*^ (0.657) | -1.462^**^ (0.527) | -1.122^**^ (0.541) |
|  |  |  |  |  |  |  |  |  |  |
| Agreeableness | -1.022 (0.955) | -1.763^**^ (0.728) | -1.326^*^ (0.730) | -1.059 (0.698) | 0.919 (0.796) | -0.921 (0.741) | -1.056 (0.714) | -0.371 (0.559) | -0.257 (0.596) |
|  |  |  |  |  |  |  |  |  |  |
| Neuroticism | 1.014 (0.775) | 0.873 (0.754) | 0.650 (0.554) | -0.283 (0.500) | 0.024 (0.577) | 0.089 (0.474) | 0.723 (0.596) | -0.061 (0.437) | -0.154 (0.404) |
|  |  |  |  |  |  |  |  |  |  |
| Education |  |  |  |  |  |  |  |  |  |
|  |  |  |  |  |  |  |  |  |  |
| Upper, post secondary | -0.770 (0.684) | 0.285 (0.847) | -0.295 (0.579) | 0.717 (0.698) | -0.001 (0.479) | -0.580 (0.404) | 0.045 (0.462) | -0.152 (0.317) | -0.281 (0.355) |
|  |  |  |  |  |  |  |  |  |  |
| Tertiary | 0.828 (0.632) | 1.024 (0.819) | 0.227 (0.606) | 0.703 (0.723) | 0.245 (0.617) | -0.308 (0.420) | 0.928^*^ (0.544) | -0.073 (0.384) | -0.651 (0.443) |
|  |  |  |  |  |  |  |  |  |  |
| Male | 1.390^**^ (0.519) | 0.729 (0.451) | 0.332 (0.415) | 0.221 (0.347) | 0.296 (0.396) | 0.639^**^ (0.313) | 0.707^*^ (0.407) | 0.510^*^ (0.283) | 0.240 (0.311) |
|  |  |  |  |  |  |  |  |  |  |
| Age | -0.049^**^ (0.015) | -0.026^**^ (0.012) | -0.032^**^ (0.013) | -0.042^**^ (0.012) | -0.008 (0.013) | -0.044^**^ (0.010) | -0.016 (0.010) | -0.032^**^ (0.008) | -0.044^**^ (0.010) |
|  |  |  |  |  |  |  |  |  |  |
| Income situation | -0.340 (0.217) | -0.474^**^ (0.182) | -0.206 (0.162) | -0.341^*^ (0.176) | -0.461^*^ (0.252) | -0.187 (0.154) | -0.274 (0.208) | -0.019 (0.127) | 0.040 (0.118) |
|  |  |  |  |  |  |  |  |  |  |
| Constant | 1.899 (2.463) | 0.596 (2.540) | -1.725 (1.954) | 2.041 (1.581) | -0.057 (1.836) | 1.070 (1.391) | -1.476 (1.933) | -0.457 (1.209) | 0.506 (1.349) |
| Pseudo *R*^2^ | 0.266 | 0.161 | 0.065 | 0.115 | 0.112 | 0.083 | 0.121 | 0.114 | 0.069 |
| Observations | 1003 | 987 | 989 | 1006 | 1020 | 978 | 993 | 981 | 966 |

*Note*: Logistic modeling coefficients with robust standard errors in parentheses. Reference category for education is “primary & lower secondary”. ^*^ *p* < 0.10, ^**^ *p* < 0.05

**AT9b**: Big Five of Personality and Self-Infection with Covid-19 in Switzerland, Spain and the United Kingdom

|  | **Switzerland** | | | **Spain** | | | **United Kingdom** | | |
| --- | --- | --- | --- | --- | --- | --- | --- | --- | --- |
| Pandemic phase | Spring 2020 | Winter 2020/2021 | Spring 2021 | Spring 2020 | Winter 2020/2021 | Spring 2021 | Spring 2020 | Winter 2020/2021 | Spring 2021 |
|  |  |  |  |  |  |  |  |  |  |
| Openness to experience | -0.101 (1.497) | 0.243 (0.762) | 0.787 (0.637) | -0.182 (0.584) | -0.920 (0.754) | 0.671 (0.545) | -2.061^*^ (1.225) | -0.901 (0.760) | -0.854 (0.521) |
|  |  |  |  |  |  |  |  |  |  |
| Extraversion | 3.305^**^ (0.858) | -0.276 (0.435) | 0.734 (0.615) | 1.127^*^ (0.669) | 1.623^**^ (0.565) | 0.415 (0.527) | 0.121 (1.020) | 0.892 (0.552) | 0.509 (0.376) |
|  |  |  |  |  |  |  |  |  |  |
| Conscientiousness | 0.633 (0.931) | -0.597 (0.582) | -0.996 (0.634) | -2.857^**^ (0.607) | -1.362^**^ (0.521) | -1.829^**^ (0.661) | -0.783 (0.915) | -0.403 (0.522) | -0.825^*^ (0.422) |
|  |  |  |  |  |  |  |  |  |  |
| Agreeableness | 0.643 (0.982) | -0.786 (0.697) | -1.094 (0.695) | 0.063 (1.364) | -0.896 (0.898) | -0.668 (0.847) | -1.510 (1.166) | -0.103 (0.691) | -0.147 (0.504) |
|  |  |  |  |  |  |  |  |  |  |
| Neuroticism | 1.449^**^ (0.635) | -0.327 (0.425) | 0.273 (0.484) | 0.734 (0.647) | -0.268 (0.461) | -0.004 (0.456) | 0.878 (1.029) | 0.712 (0.478) | 1.029^**^ (0.416) |
|  |  |  |  |  |  |  |  |  |  |
| Education |  |  |  |  |  |  |  |  |  |
|  |  |  |  |  |  |  |  |  |  |
| Upper, post secondary | -0.370 (0.713) | -1.019^**^ (0.400) | 0.256 (0.678) | 0.126 (0.518) | -0.196 (0.429) | 0.230 (0.400) | -0.443 (0.620) | 1.077^*^ (0.630) | 0.074 (0.416) |
|  |  |  |  |  |  |  |  |  |  |
| Tertiary | 0.000 (.) | -0.332 (0.380) | 0.631 (0.675) | 0.164 (0.450) | 0.078 (0.418) | 0.832^**^ (0.394) | -0.054 (0.500) | 1.216^**^ (0.599) | 0.228 (0.323) |
|  |  |  |  |  |  |  |  |  |  |
| Male | 1.338^**^ (0.644) | 0.262 (0.315) | -0.389 (0.324) | 0.523 (0.402) | -0.226 (0.329) | 0.158 (0.286) | 0.080 (0.463) | 0.686^*^ (0.365) | 1.198^**^ (0.294) |
|  |  |  |  |  |  |  |  |  |  |
| Age | -0.042^**^ (0.018) | -0.024^**^ (0.009) | -0.008 (0.009) | -0.002 (0.014) | -0.016 (0.012) | -0.005 (0.010) | -0.035^**^ (0.016) | -0.017 (0.014) | -0.026^**^ (0.008) |
|  |  |  |  |  |  |  |  |  |  |
| Income situation | -0.635^**^ (0.316) | -0.083 (0.133) | -0.108 (0.148) | -0.234 (0.208) | 0.042 (0.167) | -0.160 (0.153) | -0.238 (0.220) | -0.096 (0.170) | 0.047 (0.131) |
|  |  |  |  |  |  |  |  |  |  |
| Constant | -7.805^**^ (2.227) | 0.695 (1.507) | -1.738 (1.886) | -1.530 (2.647) | -0.013 (1.765) | -0.779 (1.716) | 2.680 (2.856) | -3.439^*^ (1.782) | -1.611 (1.407) |
| Pseudo *R*^2^ | 0.140 | 0.058 | 0.044 | 0.119 | 0.048 | 0.048 | 0.108 | 0.078 | 0.108 |
| Observations | 897 | 1120 | 985 | 992 | 1000 | 1012 | 1000 | 1029 | 984 |

*Note*: Logistic modeling coefficients with robust standard errors in parentheses. Reference category for education is “primary & lower secondary”. ^*^ *p* < 0.10, ^**^ *p* < 0.05

**AF7**: Descriptive Overview: Perceived Importance of Closing Borders


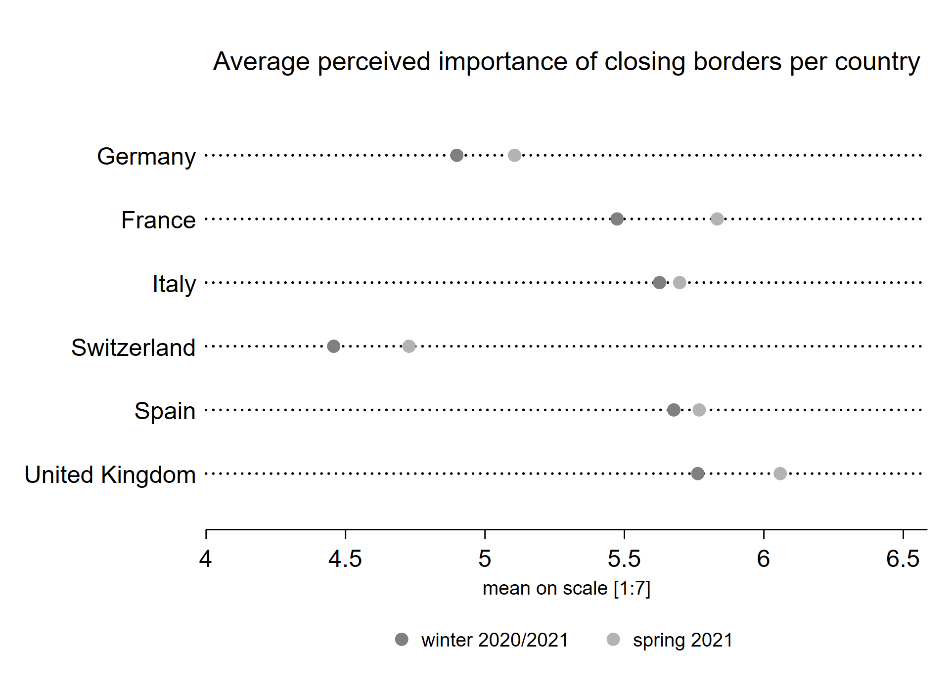


*Note*: Displayed are the mean levels of perceived importance of closing borders per country and pandemic phase.

**AT10a**: Big Five of Personality and Perceived Importance of Closing Borders in Germany, France and Italy

|  | **Germany** | | **France** | | **Italy** | |
| --- | --- | --- | --- | --- | --- | --- |
| Pandemic phase | Winter 2020/2021 | Spring 2021 | Winter 2020/2021 | Spring 2021 | Winter 2020/2021 | Spring 2021 |
|  |  |  |  |  |  |  |
| Openness to experience | 0.083 (0.316) | 0.093 (0.300) | -0.240 (0.226) | -0.381^*^ (0.199) | -0.559^**^ (0.209) | -0.441^**^ (0.190) |
|  |  |  |  |  |  |  |
| Extraversion | -0.058 (0.186) | -0.093 (0.183) | 0.039 (0.162) | -0.199 (0.154) | -0.121 (0.153) | 0.007 (0.162) |
|  |  |  |  |  |  |  |
| Conscientiousness | 0.695^**^ (0.320) | 0.528 (0.327) | 1.160^**^ (0.290) | 0.693^**^ (0.260) | 0.544^**^ (0.251) | 0.604^**^ (0.265) |
|  |  |  |  |  |  |  |
| Agreeableness | 0.055 (0.334) | 0.631^*^ (0.330) | -0.186 (0.270) | -0.009 (0.272) | 0.610^**^ (0.277) | 0.946^**^ (0.267) |
|  |  |  |  |  |  |  |
| Neuroticism | 0.336^*^ (0.185) | 0.253 (0.178) | 0.146 (0.165) | 0.124 (0.154) | 0.251 (0.171) | 0.150 (0.154) |
|  |  |  |  |  |  |  |
| Education |  |  |  |  |  |  |
|  |  |  |  |  |  |  |
| Upper, post secondary | -0.133 (0.177) | -0.232 (0.168) | -0.035 (0.145) | 0.048 (0.128) | -0.040 (0.113) | -0.073 (0.113) |
|  |  |  |  |  |  |  |
| Tertiary | -0.379^**^ (0.192) | -0.387^**^ (0.181) | -0.258 (0.163) | -0.159 (0.155) | -0.335^**^ (0.152) | -0.313^**^ (0.150) |
|  |  |  |  |  |  |  |
| Male | -0.211^*^ (0.121) | -0.151 (0.114) | -0.172 (0.111) | -0.302^**^ (0.103) | -0.429^**^ (0.103) | -0.333^**^ (0.098) |
|  |  |  |  |  |  |  |
| Age | -0.001 (0.004) | 0.003 (0.003) | 0.014^**^ (0.004) | 0.018^**^ (0.004) | 0.006^*^ (0.003) | 0.008^**^ (0.003) |
|  |  |  |  |  |  |  |
| Income situation | -0.022 (0.058) | -0.017 (0.057) | 0.041 (0.055) | 0.035 (0.057) | -0.051 (0.057) | 0.025 (0.050) |
|  |  |  |  |  |  |  |
| Constant | 3.973^**^ (0.778) | 3.590^**^ (0.781) | 3.661^**^ (0.638) | 4.650^**^ (0.558) | 4.766^**^ (0.637) | 3.841^**^ (0.562) |
| *R*^2^ | 0.023 | 0.025 | 0.057 | 0.065 | 0.065 | 0.081 |
| Adjusted *R*^2^ | 0.013 | 0.015 | 0.048 | 0.055 | 0.056 | 0.072 |
| Observations | 989 | 990 | 1022 | 983 | 981 | 977 |

*Note*: Linear regression coefficients with robust standard errors in parentheses. Reference category for education is “primary & lower secondary”. ^*^ *p* < 0.10, ^**^ *p* < 0.05

**AT10b**: Big Five of Personality and Perceived Importance of Closing Borders in Switzerland, Spain and the United Kingdom

|  | **Switzerland** | | **Spain** | | **United Kingdom** | |
| --- | --- | --- | --- | --- | --- | --- |
| Pandemic phase | Winter 2020/2021 | Spring 2021 | Winter 2020/2021 | Spring 2021 | Winter 2020/2021 | Spring 2021 |
|  |  |  |  |  |  |  |
| Openness to experience | -0.158 (0.278) | -0.147 (0.186) | 0.079 (0.209) | -0.147 (0.186) | -0.118 (0.222) | -0.096 (0.177) |
|  |  |  |  |  |  |  |
| Extraversion | 0.046 (0.191) | 0.346^**^ (0.147) | -0.061 (0.174) | 0.346^**^ (0.147) | 0.058 (0.137) | -0.142 (0.116) |
|  |  |  |  |  |  |  |
| Conscientiousness | 0.516 (0.321) | 1.003^**^ (0.232) | 0.885^**^ (0.270) | 1.003^**^ (0.232) | 0.419^**^ (0.197) | 0.583^**^ (0.205) |
|  |  |  |  |  |  |  |
| Agreeableness | 0.303 (0.312) | -0.176 (0.248) | 0.471^*^ (0.274) | -0.176 (0.248) | 0.526^**^ (0.247) | 0.270 (0.207) |
|  |  |  |  |  |  |  |
| Neuroticism | 0.488^**^ (0.164) | 0.004 (0.139) | 0.030 (0.144) | 0.004 (0.139) | 0.315^**^ (0.145) | 0.121 (0.103) |
|  |  |  |  |  |  |  |
| Education |  |  |  |  |  |  |
|  |  |  |  |  |  |  |
| Upper, post secondary | 0.240 (0.188) | 0.044 (0.116) | -0.332^**^ (0.126) | 0.044 (0.116) | 0.052 (0.124) | 0.238^**^ (0.119) |
|  |  |  |  |  |  |  |
| Tertiary | -0.248 (0.191) | -0.275^**^ (0.124) | -0.568^**^ (0.117) | -0.275^**^ (0.124) | -0.075 (0.132) | 0.144 (0.112) |
|  |  |  |  |  |  |  |
| Male | 0.013 (0.112) | -0.333^**^ (0.092) | -0.320^**^ (0.094) | -0.333^**^ (0.092) | -0.178^*^ (0.093) | -0.245^**^ (0.093) |
|  |  |  |  |  |  |  |
| Age | 0.001 (0.003) | 0.010^**^ (0.003) | 0.005 (0.003) | 0.010^**^ (0.003) | 0.013^**^ (0.003) | 0.024^**^ (0.003) |
|  |  |  |  |  |  |  |
| Income situation | 0.024 (0.052) | -0.045 (0.049) | 0.094^*^ (0.049) | -0.045 (0.049) | -0.033 (0.043) | 0.026 (0.041) |
|  |  |  |  |  |  |  |
| Constant | 2.988^**^ (0.663) | 4.378^**^ (0.538) | 3.850^**^ (0.594) | 4.378^**^ (0.538) | 3.970^**^ (0.546) | 3.900^**^ (0.499) |
| *R*^2^ | 0.030 | 0.071 | 0.063 | 0.071 | 0.045 | 0.113 |
| Adjusted *R*^2^ | 0.022 | 0.062 | 0.054 | 0.062 | 0.036 | 0.104 |
| Observations | 1120 | 1016 | 1002 | 1016 | 1029 | 986 |

*Note*: Linear regression coefficients with robust standard errors in parentheses. Reference category for education is “primary & lower secondary”. ^*^ *p* < 0.10, ^**^ *p* < 0.05

**AF8**: Descriptive Overview: Covid-19 Intolerance


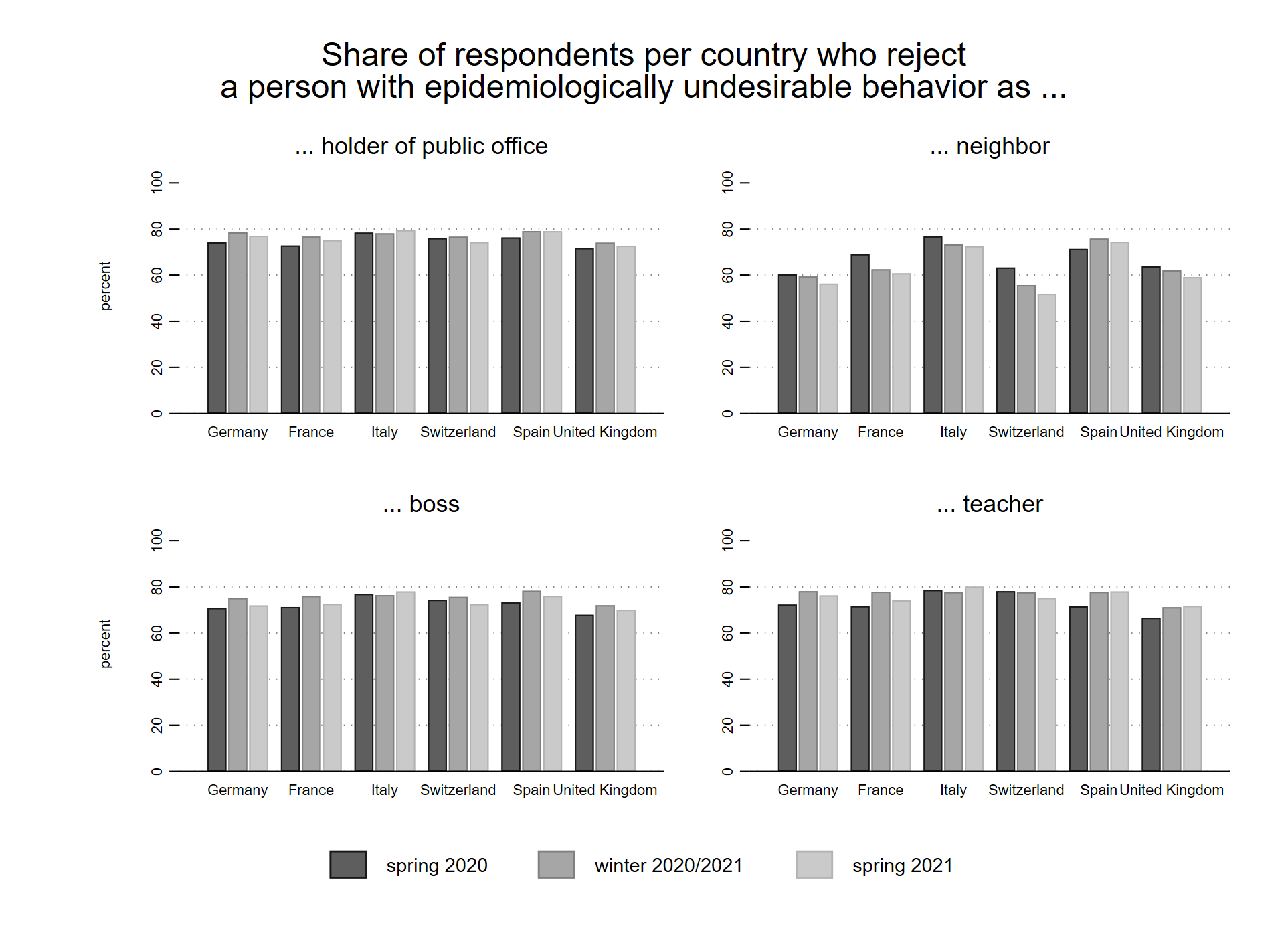


*Note*: The proportions displayed equal the share of respondents per country and pandemic phase who reject a person with epidemiologically undesirable

behavior as public office holders, neighbors, bosses, or teachers.

**AT11a**: Big Five of Personality and Covid-19-Intolerance (“Public Office”) in Germany, France and Italy

|  | **Germany** | | | **France** | | | **Italy** | | |
| --- | --- | --- | --- | --- | --- | --- | --- | --- | --- |
| Pandemic phase | Spring 2020 | Winter 2020/2021 | Spring 2021 | Spring 2020 | Winter 2020/2021 | Spring 2021 | Spring 2020 | Winter 2020/2021 | Spring 2021 |
|  |  |  |  |  |  |  |  |  |  |
| Openness to experience | 0.861^**^ (0.352) | 0.423 (0.396) | 0.270 (0.387) | 0.387 (0.321) | 0.850^**^ (0.321) | 0.036 (0.343) | 0.311 (0.339) | 0.747^**^ (0.317) | 0.076 (0.365) |
|  |  |  |  |  |  |  |  |  |  |
| Extraversion | -0.563^**^ (0.259) | -0.370 (0.267) | -0.702^**^ (0.278) | -0.442^*^ (0.247) | -0.093 (0.247) | -0.637^**^ (0.263) | -0.433^*^ (0.234) | -0.425 (0.276) | -0.554^**^ (0.282) |
|  |  |  |  |  |  |  |  |  |  |
| Conscientiousness | 0.257 (0.351) | 0.626 (0.385) | 0.379 (0.413) | 0.858^**^ (0.337) | 0.684^**^ (0.335) | 1.487^**^ (0.401) | 0.086 (0.360) | 1.040^**^ (0.366) | 1.033^**^ (0.353) |
|  |  |  |  |  |  |  |  |  |  |
| Agreeableness | 0.436 (0.374) | 0.659 (0.413) | 0.827^**^ (0.393) | -0.261 (0.379) | 0.502 (0.362) | -0.411 (0.450) | 0.201 (0.368) | 0.961^**^ (0.401) | 1.353^**^ (0.369) |
|  |  |  |  |  |  |  |  |  |  |
| Neuroticism | 0.161 (0.214) | -0.303 (0.248) | 0.062 (0.248) | 0.115 (0.224) | -0.459^*^ (0.248) | -0.133 (0.244) | -0.064 (0.232) | -0.126 (0.248) | -0.247 (0.265) |
|  |  |  |  |  |  |  |  |  |  |
| Education |  |  |  |  |  |  |  |  |  |
|  |  |  |  |  |  |  |  |  |  |
| Upper, post secondary | -0.096 (0.220) | 0.064 (0.229) | 0.329 (0.224) | 0.097 (0.197) | 0.227 (0.201) | 0.326 (0.212) | 0.003 (0.180) | 0.051 (0.183) | 0.229 (0.211) |
|  |  |  |  |  |  |  |  |  |  |
| Tertiary | 0.022 (0.241) | 0.462^*^ (0.263) | 0.699^**^ (0.258) | 0.760^**^ (0.230) | 0.642^**^ (0.232) | 0.433^*^ (0.235) | 0.363 (0.246) | 0.126 (0.233) | -0.067 (0.242) |
|  |  |  |  |  |  |  |  |  |  |
| Male | -0.203 (0.153) | -0.320^*^ (0.183) | -0.331^*^ (0.170) | -0.015 (0.154) | -0.193 (0.167) | -0.357^**^ (0.162) | -0.167 (0.164) | -0.187 (0.164) | -0.300^*^ (0.174) |
|  |  |  |  |  |  |  |  |  |  |
| Age | 0.006 (0.005) | -0.001 (0.005) | 0.012^**^ (0.005) | 0.017^**^ (0.005) | 0.028^**^ (0.005) | 0.022^**^ (0.006) | 0.018^**^ (0.005) | 0.009^*^ (0.005) | 0.019^**^ (0.006) |
|  |  |  |  |  |  |  |  |  |  |
| Income situation | 0.045 (0.069) | 0.214^**^ (0.082) | 0.122 (0.080) | 0.099 (0.077) | 0.141^*^ (0.081) | 0.057 (0.085) | 0.064 (0.086) | 0.023 (0.080) | 0.184^**^ (0.081) |
|  |  |  |  |  |  |  |  |  |  |
| Constant | -0.760 (0.849) | -0.924 (1.035) | -1.188 (0.967) | -1.363^*^ (0.772) | -2.734^**^ (0.824) | -0.949 (0.847) | 0.059 (0.792) | -1.951^**^ (0.899) | -2.280^**^ (0.913) |
| Pseudo *R*^2^ | 0.020 | 0.040 | 0.037 | 0.041 | 0.067 | 0.049 | 0.023 | 0.055 | 0.091 |
| Observations | 1003 | 989 | 990 | 1007 | 1022 | 983 | 993 | 981 | 977 |

*Note*: Logistic modeling coefficients with robust standard errors in parentheses. Reference category for education is “primary & lower secondary”. ^*^ *p* < 0.10, ^**^ *p* < 0.05

**AT11b**: Big Five of Personality and Covid-19-Intolerance (“Public Office”) in Switzerland, Spain and the United Kingdom

|  | **Switzerland** | | | **Spain** | | | **United Kingdom** | | |
| --- | --- | --- | --- | --- | --- | --- | --- | --- | --- |
| Pandemic phase | Spring 2020 | Winter 2020/2021 | Spring 2021 | Spring 2020 | Winter 2020/2021 | Spring 2021 | Spring 2020 | Winter 2020/2021 | Spring 2021 |
|  |  |  |  |  |  |  |  |  |  |
| Openness to experience | -0.139 (0.422) | 0.438 (0.336) | 0.015 (0.362) | 0.526^*^ (0.309) | 1.162^**^ (0.330) | 0.712^**^ (0.323) | 0.199 (0.467) | 0.100 (0.319) | -0.036 (0.309) |
|  |  |  |  |  |  |  |  |  |  |
| Extraversion | -0.467^*^ (0.267) | -0.487^*^ (0.253) | -0.106 (0.262) | -0.342 (0.259) | -0.861^**^ (0.331) | -0.055 (0.280) | -0.878^**^ (0.327) | -0.299 (0.222) | -0.291 (0.213) |
|  |  |  |  |  |  |  |  |  |  |
| Conscientiousness | 0.843^**^ (0.423) | 0.928^**^ (0.375) | 0.885^**^ (0.381) | 0.371 (0.337) | 1.972^**^ (0.387) | 1.810^**^ (0.390) | 0.893^*^ (0.462) | 0.691^**^ (0.320) | 0.437 (0.312) |
|  |  |  |  |  |  |  |  |  |  |
| Agreeableness | 0.720^*^ (0.410) | 0.546 (0.386) | 2.247^**^ (0.411) | 0.547 (0.439) | 0.209 (0.467) | 0.477 (0.483) | -0.385 (0.482) | 0.498 (0.331) | 0.227 (0.330) |
|  |  |  |  |  |  |  |  |  |  |
| Neuroticism | 0.484^**^ (0.239) | -0.203 (0.221) | 0.787^**^ (0.237) | 0.297 (0.243) | -0.232 (0.262) | -0.295 (0.264) | -0.038 (0.320) | 0.350 (0.220) | 0.325^*^ (0.196) |
|  |  |  |  |  |  |  |  |  |  |
| Education |  |  |  |  |  |  |  |  |  |
|  |  |  |  |  |  |  |  |  |  |
| Upper, post secondary | 0.672^**^ (0.252) | 0.182 (0.241) | 0.330 (0.284) | 0.241 (0.202) | 0.043 (0.206) | -0.118 (0.212) | 0.128 (0.202) | 0.472^**^ (0.221) | 0.269 (0.218) |
|  |  |  |  |  |  |  |  |  |  |
| Tertiary | 0.634^**^ (0.264) | 0.295 (0.247) | 0.548^*^ (0.290) | 0.407^**^ (0.185) | 0.466^**^ (0.211) | 0.350 (0.213) | 0.475^**^ (0.175) | 0.533^**^ (0.209) | 0.356^*^ (0.188) |
|  |  |  |  |  |  |  |  |  |  |
| Male | -0.006 (0.167) | -0.388^**^ (0.155) | 0.215 (0.158) | 0.098 (0.154) | 0.210 (0.166) | 0.215 (0.165) | -0.169 (0.157) | -0.182 (0.166) | -0.102 (0.159) |
|  |  |  |  |  |  |  |  |  |  |
| Age | 0.020^**^ (0.005) | 0.010^**^ (0.004) | 0.019^**^ (0.005) | 0.017^**^ (0.005) | 0.014^**^ (0.006) | 0.018^**^ (0.006) | 0.022^**^ (0.005) | 0.037^**^ (0.006) | 0.023^**^ (0.005) |
|  |  |  |  |  |  |  |  |  |  |
| Income situation | 0.128^*^ (0.071) | 0.139^**^ (0.069) | 0.119 (0.075) | 0.096 (0.074) | 0.009 (0.075) | 0.072 (0.084) | 0.039 (0.069) | 0.008 (0.070) | 0.126^*^ (0.070) |
|  |  |  |  |  |  |  |  |  |  |
| Constant | -2.531^**^ (0.904) | -1.532^*^ (0.831) | -5.391^**^ (0.991) | -1.806^*^ (0.927) | -2.680^**^ (0.999) | -3.316^**^ (1.013) | -0.302 (1.115) | -2.569^**^ (0.854) | -1.484^*^ (0.771) |
| Pseudo *R*^2^ | 0.046 | 0.049 | 0.067 | 0.028 | 0.076 | 0.067 | 0.040 | 0.056 | 0.035 |
| Observations | 990 | 1120 | 990 | 992 | 1002 | 1016 | 1000 | 1029 | 986 |

Note: Logistic modeling coefficients with robust standard errors in parentheses. Reference category for education is “primary & lower secondary”. ^*^ *p* < 0.10, ^**^ *p* < 0.05

**AT12a**: Big Five of Personality and Covid-19-Intolerance (“Neighbor”) in Germany, France and Italy

|  | **Germany** | | | **France** | | | **Italy** | | |
| --- | --- | --- | --- | --- | --- | --- | --- | --- | --- |
| Pandemic phase | Spring 2020 | Winter 2020/2021 | Spring 2021 | Spring 2020 | Winter 2020/2021 | Spring 2021 | Spring 2020 | Winter 2020/2021 | Spring 2021 |
|  |  |  |  |  |  |  |  |  |  |
| Openness to experience | 0.276 (0.319) | 0.064 (0.349) | 0.436 (0.320) | 0.341 (0.306) | 0.341 (0.301) | -0.282 (0.287) | 0.201 (0.323) | 0.439 (0.301) | -0.499 (0.337) |
|  |  |  |  |  |  |  |  |  |  |
| Extraversion | -0.169 (0.227) | 0.273 (0.215) | -0.401^*^ (0.210) | -0.038 (0.223) | -0.107 (0.213) | -0.228 (0.205) | -0.329 (0.230) | -0.377 (0.241) | -0.046 (0.239) |
|  |  |  |  |  |  |  |  |  |  |
| Conscientiousness | 0.242 (0.328) | 0.507 (0.342) | 0.256 (0.351) | 0.833^**^ (0.325) | 0.821^**^ (0.337) | 0.773^**^ (0.345) | -0.033 (0.367) | 0.577^*^ (0.338) | 0.883^**^ (0.329) |
|  |  |  |  |  |  |  |  |  |  |
| Agreeableness | 0.341 (0.346) | 0.270 (0.367) | -0.076 (0.353) | -0.341 (0.362) | 0.740^**^ (0.347) | -0.399 (0.372) | 0.148 (0.355) | 0.378 (0.371) | 1.096^**^ (0.345) |
|  |  |  |  |  |  |  |  |  |  |
| Neuroticism | -0.020 (0.188) | 0.393^*^ (0.203) | 0.356^*^ (0.198) | -0.008 (0.218) | -0.008 (0.213) | -0.158 (0.213) | -0.205 (0.229) | 0.159 (0.232) | 0.054 (0.232) |
|  |  |  |  |  |  |  |  |  |  |
| Education |  |  |  |  |  |  |  |  |  |
|  |  |  |  |  |  |  |  |  |  |
| Upper, post secondary | 0.131 (0.192) | 0.402^**^ (0.196) | -0.114 (0.198) | 0.192 (0.189) | 0.089 (0.177) | -0.078 (0.185) | 0.075 (0.173) | 0.123 (0.167) | 0.180 (0.183) |
|  |  |  |  |  |  |  |  |  |  |
| Tertiary | 0.355^*^ (0.211) | 0.474^**^ (0.218) | -0.000 (0.217) | 0.413^*^ (0.214) | 0.391^*^ (0.203) | 0.080 (0.207) | 0.315 (0.233) | 0.195 (0.214) | 0.115 (0.219) |
|  |  |  |  |  |  |  |  |  |  |
| Male | -0.162 (0.136) | 0.209 (0.146) | 0.024 (0.142) | -0.068 (0.148) | 0.096 (0.142) | -0.120 (0.139) | -0.185 (0.159) | -0.095 (0.150) | -0.257^*^ (0.154) |
|  |  |  |  |  |  |  |  |  |  |
| Age | 0.001 (0.004) | 0.010^**^ (0.004) | 0.016^**^ (0.004) | 0.016^**^ (0.005) | 0.020^**^ (0.005) | 0.015^**^ (0.005) | 0.012^**^ (0.005) | 0.014^**^ (0.005) | 0.017^**^ (0.005) |
|  |  |  |  |  |  |  |  |  |  |
| Income situation | -0.068 (0.064) | 0.132^*^ (0.068) | 0.072 (0.064) | 0.158^**^ (0.072) | 0.054 (0.068) | -0.011 (0.071) | 0.081 (0.082) | 0.028 (0.072) | 0.169^**^ (0.072) |
|  |  |  |  |  |  |  |  |  |  |
| Constant | -0.448 (0.753) | -2.723^**^ (0.877) | -1.373^*^ (0.823) | -1.661^**^ (0.756) | -3.150^**^ (0.762) | -0.016 (0.731) | 0.577 (0.766) | -1.246 (0.776) | -2.247^**^ (0.817) |
| Pseudo *R*^2^ | 0.009 | 0.018 | 0.020 | 0.029 | 0.040 | 0.017 | 0.014 | 0.025 | 0.056 |
| Observations | 1003 | 989 | 990 | 1007 | 1022 | 983 | 993 | 981 | 977 |

*Note*: Logistic modeling coefficients with robust standard errors in parentheses. Reference category for education is “primary & lower secondary”. ^*^ *p* < 0.10, ^**^ *p* < 0.05

**AT12b**: Big Five of Personality and Covid-19-Intolerance (“Neighbor”) in Switzerland, Spain and the United Kingdom

|  | **Switzerland** | | | **Spain** | | | **United Kingdom** | | |
| --- | --- | --- | --- | --- | --- | --- | --- | --- | --- |
| Pandemic phase | Spring 2020 | Winter 2020/2021 | Spring 2021 | Spring 2020 | Winter 2020/2021 | Spring 2021 | Spring 2020 | Winter 2020/2021 | Spring 2021 |
|  |  |  |  |  |  |  |  |  |  |
| Openness to experience | -0.369 (0.358) | -0.503^*^ (0.299) | -0.630^**^ (0.301) | 0.371 (0.297) | 0.964^**^ (0.317) | 0.649^**^ (0.300) | 0.202 (0.438) | 0.246 (0.282) | 0.192 (0.286) |
|  |  |  |  |  |  |  |  |  |  |
| Extraversion | -0.054 (0.218) | -0.291 (0.197) | 0.341 (0.212) | -0.073 (0.245) | -0.389 (0.295) | 0.036 (0.256) | -0.769^**^ (0.300) | -0.001 (0.190) | -0.095 (0.185) |
|  |  |  |  |  |  |  |  |  |  |
| Conscientiousness | 0.434 (0.366) | -0.269 (0.333) | 0.560^*^ (0.332) | 0.646^**^ (0.329) | 1.577^**^ (0.368) | 1.265^**^ (0.369) | 1.458^**^ (0.464) | 0.209 (0.292) | 0.106 (0.303) |
|  |  |  |  |  |  |  |  |  |  |
| Agreeableness | 0.294 (0.345) | 0.360 (0.321) | 0.345 (0.358) | 0.481 (0.407) | 0.126 (0.439) | 0.677 (0.414) | -0.011 (0.460) | 0.207 (0.309) | -0.228 (0.317) |
|  |  |  |  |  |  |  |  |  |  |
| Neuroticism | 0.589^**^ (0.203) | 0.121 (0.186) | 0.499^**^ (0.200) | 0.411^*^ (0.221) | -0.218 (0.260) | -0.070 (0.236) | 0.466 (0.296) | 0.221 (0.184) | 0.310^*^ (0.179) |
|  |  |  |  |  |  |  |  |  |  |
| Education |  |  |  |  |  |  |  |  |  |
|  |  |  |  |  |  |  |  |  |  |
| Upper, post secondary | 0.427^*^ (0.241) | -0.059 (0.205) | -0.037 (0.260) | 0.172 (0.189) | 0.011 (0.196) | -0.224 (0.194) | 0.167 (0.194) | 0.187 (0.191) | -0.024 (0.193) |
|  |  |  |  |  |  |  |  |  |  |
| Tertiary | 0.331 (0.248) | 0.094 (0.209) | -0.040 (0.265) | 0.207 (0.172) | 0.383^*^ (0.199) | 0.299 (0.196) | 0.335^**^ (0.162) | 0.403^**^ (0.182) | 0.196 (0.171) |
|  |  |  |  |  |  |  |  |  |  |
| Male | -0.011 (0.144) | -0.177 (0.128) | 0.202 (0.136) | 0.044 (0.145) | 0.129 (0.155) | 0.071 (0.154) | 0.092 (0.143) | 0.018 (0.143) | 0.403^**^ (0.142) |
|  |  |  |  |  |  |  |  |  |  |
| Age | 0.017^**^ (0.004) | 0.014^**^ (0.004) | 0.010^**^ (0.004) | 0.006 (0.005) | 0.015^**^ (0.005) | 0.022^**^ (0.005) | 0.014^**^ (0.005) | 0.025^**^ (0.005) | 0.011^**^ (0.004) |
|  |  |  |  |  |  |  |  |  |  |
| Income situation | 0.016 (0.064) | 0.042 (0.059) | 0.051 (0.064) | 0.169^**^ (0.072) | -0.002 (0.073) | 0.045 (0.078) | 0.010 (0.066) | -0.028 (0.062) | 0.105^*^ (0.063) |
|  |  |  |  |  |  |  |  |  |  |
| Constant | -1.561^**^ (0.782) | 0.240 (0.726) | -1.811^**^ (0.827) | -2.115^**^ (0.848) | -2.508^**^ (0.958) | -3.336^**^ (0.911) | -2.090^**^ (1.041) | -1.836^**^ (0.713) | -1.022 (0.734) |
| Pseudo *R*^2^ | 0.023 | 0.015 | 0.015 | 0.019 | 0.053 | 0.052 | 0.030 | 0.023 | 0.021 |
| Observations | 990 | 1120 | 990 | 992 | 1002 | 1016 | 1000 | 1029 | 986 |

*Note*: Logistic modeling coefficients with robust standard errors in parentheses. Reference category for education is “primary & lower secondary”. ^*^ *p* < 0.10, ^**^ *p* < 0.05

**AT13a**: Big Five of Personality and Covid-19-Intolerance (“Boss”) in Germany, France and Italy

|  | **Germany** | | | **France** | | | **Italy** | | |
| --- | --- | --- | --- | --- | --- | --- | --- | --- | --- |
| Pandemic phase | Spring 2020 | Winter 2020/2021 | Spring 2021 | Spring 2020 | Winter 2020/2021 | Spring 2021 | Spring 2020 | Winter 2020/2021 | Spring 2021 |
|  |  |  |  |  |  |  |  |  |  |
| Openness to experience | 0.599^*^ (0.339) | 0.108 (0.384) | 0.448 (0.367) | 0.518 (0.331) | 1.017^**^ (0.323) | -0.175 (0.326) | 0.193 (0.346) | 0.502 (0.313) | -0.280 (0.362) |
|  |  |  |  |  |  |  |  |  |  |
| Extraversion | -0.379 (0.256) | 0.194 (0.240) | -0.853^**^ (0.252) | -0.371 (0.243) | -0.164 (0.250) | -0.826^**^ (0.251) | -0.410^*^ (0.236) | -0.499^*^ (0.274) | -0.271 (0.267) |
|  |  |  |  |  |  |  |  |  |  |
| Conscientiousness | 0.221 (0.344) | 0.626^*^ (0.372) | 0.484 (0.383) | 1.406^**^ (0.354) | 0.694^**^ (0.333) | 0.740^**^ (0.364) | 0.552 (0.359) | 1.321^**^ (0.367) | 1.112^**^ (0.351) |
|  |  |  |  |  |  |  |  |  |  |
| Agreeableness | 0.748^**^ (0.364) | 0.976^**^ (0.408) | 0.026 (0.384) | -0.437 (0.390) | 0.505 (0.353) | 0.301 (0.408) | 0.149 (0.361) | 0.541 (0.405) | 1.141^**^ (0.359) |
|  |  |  |  |  |  |  |  |  |  |
| Neuroticism | 0.292 (0.205) | -0.184 (0.231) | -0.137 (0.235) | 0.002 (0.235) | -0.326 (0.249) | -0.057 (0.237) | -0.147 (0.235) | 0.008 (0.253) | 0.000 (0.249) |
|  |  |  |  |  |  |  |  |  |  |
| Education |  |  |  |  |  |  |  |  |  |
|  |  |  |  |  |  |  |  |  |  |
| Upper, post secondary | 0.169 (0.205) | 0.398^*^ (0.212) | 0.228 (0.212) | 0.242 (0.192) | 0.306 (0.201) | 0.185 (0.206) | -0.050 (0.176) | 0.140 (0.176) | 0.372^*^ (0.202) |
|  |  |  |  |  |  |  |  |  |  |
| Tertiary | 0.350 (0.227) | 0.770^**^ (0.245) | 0.404^*^ (0.238) | 0.845^**^ (0.223) | 0.509^**^ (0.230) | 0.316 (0.224) | 0.194 (0.234) | 0.246 (0.231) | 0.259 (0.236) |
|  |  |  |  |  |  |  |  |  |  |
| Male | -0.196 (0.147) | -0.188 (0.172) | -0.355^**^ (0.158) | -0.087 (0.153) | -0.181 (0.165) | -0.495^**^ (0.156) | -0.199 (0.160) | -0.328^**^ (0.162) | -0.350^**^ (0.168) |
|  |  |  |  |  |  |  |  |  |  |
| Age | 0.008^*^ (0.005) | 0.004 (0.005) | 0.015^**^ (0.005) | 0.015^**^ (0.005) | 0.019^**^ (0.005) | 0.023^**^ (0.005) | 0.018^**^ (0.005) | 0.012^**^ (0.005) | 0.024^**^ (0.006) |
|  |  |  |  |  |  |  |  |  |  |
| Income situation | 0.021 (0.070) | 0.249^**^ (0.079) | 0.087 (0.074) | 0.112 (0.076) | 0.189^**^ (0.081) | 0.079 (0.080) | 0.121 (0.083) | 0.154^*^ (0.081) | 0.084 (0.077) |
|  |  |  |  |  |  |  |  |  |  |
| Constant | -1.496^*^ (0.801) | -2.482^**^ (0.995) | -0.374 (0.915) | -2.020^**^ (0.830) | -2.780^**^ (0.831) | -0.556 (0.803) | -0.465 (0.777) | -2.136^**^ (0.908) | -2.435^**^ (0.880) |
| Pseudo *R*^2^ | 0.020 | 0.048 | 0.033 | 0.054 | 0.057 | 0.047 | 0.030 | 0.063 | 0.078 |
| Observations | 1003 | 989 | 990 | 1007 | 1022 | 983 | 993 | 981 | 977 |

*Note*: Logistic modeling coefficients with robust standard errors in parentheses. Reference category for education is “primary & lower secondary”. ^*^ *p* < 0.10, ^**^ *p* < 0.05

**AT13b**: Big Five of Personality and Covid-19-Intolerance (“Boss”) in Switzerland, Spain and the United Kingdom

|  | **Switzerland** | | | **Spain** | | | **United Kingdom** | | |
| --- | --- | --- | --- | --- | --- | --- | --- | --- | --- |
| Pandemic phase | Spring 2020 | Winter 2020/2021 | Spring 2021 | Spring 2020 | Winter 2020/2021 | Spring 2021 | Spring 2020 | Winter 2020/2021 | Spring 2021 |
|  |  |  |  |  |  |  |  |  |  |
| Openness to experience | -0.195 (0.410) | 0.527 (0.333) | 0.066 (0.347) | 0.336 (0.304) | 1.041^**^ (0.329) | 0.810^**^ (0.297) | 0.519 (0.444) | 0.197 (0.304) | 0.187 (0.300) |
|  |  |  |  |  |  |  |  |  |  |
| Extraversion | -0.314 (0.251) | -0.219 (0.242) | 0.098 (0.248) | -0.098 (0.253) | -0.908^**^ (0.318) | 0.070 (0.263) | -1.203^**^ (0.315) | -0.290 (0.210) | -0.060 (0.204) |
|  |  |  |  |  |  |  |  |  |  |
| Conscientiousness | 0.384 (0.396) | 0.260 (0.380) | 1.295^**^ (0.365) | 0.576^*^ (0.341) | 1.759^**^ (0.372) | 1.596^**^ (0.375) | 0.801^*^ (0.451) | 0.167 (0.304) | 0.052 (0.303) |
|  |  |  |  |  |  |  |  |  |  |
| Agreeableness | 0.846^**^ (0.381) | 0.756^**^ (0.371) | 1.303^**^ (0.409) | 0.396 (0.424) | 0.488 (0.453) | 0.369 (0.437) | 0.092 (0.463) | 0.716^**^ (0.328) | 0.515 (0.329) |
|  |  |  |  |  |  |  |  |  |  |
| Neuroticism | 0.454^**^ (0.226) | 0.002 (0.224) | 0.434^*^ (0.232) | 0.217 (0.237) | -0.042 (0.263) | -0.169 (0.251) | 0.070 (0.309) | 0.288 (0.206) | 0.434^**^ (0.192) |
|  |  |  |  |  |  |  |  |  |  |
| Education |  |  |  |  |  |  |  |  |  |
|  |  |  |  |  |  |  |  |  |  |
| Upper, post secondary | 0.793^**^ (0.248) | -0.152 (0.250) | 0.527^*^ (0.281) | 0.166 (0.194) | -0.025 (0.204) | -0.338^*^ (0.200) | 0.146 (0.199) | 0.582^**^ (0.213) | 0.070 (0.206) |
|  |  |  |  |  |  |  |  |  |  |
| Tertiary | 0.757^**^ (0.258) | -0.056 (0.253) | 0.641^**^ (0.290) | 0.281 (0.178) | 0.331 (0.206) | 0.320 (0.206) | 0.345^**^ (0.168) | 0.750^**^ (0.202) | 0.297 (0.182) |
|  |  |  |  |  |  |  |  |  |  |
| Male | -0.064 (0.161) | -0.312^**^ (0.149) | -0.010 (0.155) | 0.040 (0.149) | 0.127 (0.162) | 0.202 (0.160) | -0.046 (0.150) | -0.192 (0.159) | 0.046 (0.154) |
|  |  |  |  |  |  |  |  |  |  |
| Age | 0.016^**^ (0.005) | 0.006 (0.004) | 0.019^**^ (0.005) | 0.014^**^ (0.005) | 0.011^*^ (0.006) | 0.019^**^ (0.006) | 0.018^**^ (0.005) | 0.033^**^ (0.006) | 0.018^**^ (0.005) |
|  |  |  |  |  |  |  |  |  |  |
| Income situation | 0.069 (0.071) | 0.177^**^ (0.070) | 0.168^**^ (0.074) | 0.095 (0.074) | -0.055 (0.075) | -0.002 (0.079) | 0.054 (0.067) | 0.010 (0.067) | 0.120^*^ (0.067) |
|  |  |  |  |  |  |  |  |  |  |
| Constant | -1.982^**^ (0.849) | -1.169 (0.811) | -4.985^**^ (0.937) | -1.790^**^ (0.900) | -2.320^**^ (0.984) | -3.218^**^ (0.924) | -0.960 (1.059) | -2.262^**^ (0.796) | -1.825^**^ (0.757) |
| Pseudo *R*^2^ | 0.034 | 0.026 | 0.063 | 0.022 | 0.056 | 0.063 | 0.037 | 0.043 | 0.024 |
| Observations | 990 | 1120 | 990 | 992 | 1002 | 1016 | 1000 | 1029 | 986 |

*Note*: Logistic modeling coefficients with robust standard errors in parentheses. Reference category for education is “primary & lower secondary”. ^*^ *p* < 0.10, ^**^ *p* < 0.05

**AT14a**: Big Five of Personality and Covid-19-Intolerance (“Teacher”) in Germany, France and Italy

|  | **Germany** | | | **France** | | | **Italy** | | |
| --- | --- | --- | --- | --- | --- | --- | --- | --- | --- |
| Pandemic phase | Spring 2020 | Winter 2020/2021 | Spring 2021 | Spring 2020 | Winter 2020/2021 | Spring 2021 | Spring 2020 | Winter 2020/2021 | Spring 2021 |
|  |  |  |  |  |  |  |  |  |  |
| Openness to experience | 0.917^**^ (0.355) | -0.064 (0.398) | 0.504 (0.376) | 0.649^**^ (0.325) | 0.998^**^ (0.307) | -0.044 (0.336) | 0.027 (0.345) | 0.384 (0.309) | -0.348 (0.387) |
|  |  |  |  |  |  |  |  |  |  |
| Extraversion | -0.642^**^ (0.251) | 0.154 (0.245) | -0.957^**^ (0.285) | -0.555^**^ (0.244) | -0.106 (0.247) | -0.563^**^ (0.251) | -0.274 (0.237) | -0.528^*^ (0.274) | -0.402 (0.280) |
|  |  |  |  |  |  |  |  |  |  |
| Conscientiousness | 0.304 (0.353) | 0.665^*^ (0.380) | 0.391 (0.408) | 0.753^**^ (0.331) | 0.136 (0.331) | 1.312^**^ (0.383) | 0.520 (0.362) | 1.274^**^ (0.372) | 1.143^**^ (0.355) |
|  |  |  |  |  |  |  |  |  |  |
| Agreeableness | 0.628^*^ (0.370) | 0.499 (0.409) | 0.429 (0.392) | -0.081 (0.371) | 1.279^**^ (0.380) | 0.173 (0.420) | -0.262 (0.349) | 0.465 (0.411) | 1.018^**^ (0.373) |
|  |  |  |  |  |  |  |  |  |  |
| Neuroticism | 0.257 (0.210) | -0.285 (0.239) | -0.097 (0.251) | 0.120 (0.222) | -0.202 (0.246) | 0.043 (0.237) | -0.090 (0.235) | 0.042 (0.250) | -0.364 (0.273) |
|  |  |  |  |  |  |  |  |  |  |
| Education |  |  |  |  |  |  |  |  |  |
|  |  |  |  |  |  |  |  |  |  |
| Upper, post secondary | 0.022 (0.213) | 0.287 (0.220) | 0.155 (0.225) | 0.326^*^ (0.191) | 0.177 (0.201) | 0.261 (0.212) | -0.072 (0.181) | 0.263 (0.182) | 0.275 (0.212) |
|  |  |  |  |  |  |  |  |  |  |
| Tertiary | 0.082 (0.232) | 0.630^**^ (0.253) | 0.425^*^ (0.256) | 0.928^**^ (0.225) | 0.632^**^ (0.233) | 0.434^*^ (0.234) | 0.211 (0.245) | 0.201 (0.229) | 0.022 (0.244) |
|  |  |  |  |  |  |  |  |  |  |
| Male | -0.277^*^ (0.150) | -0.336^*^ (0.183) | -0.448^**^ (0.170) | -0.232 (0.155) | -0.142 (0.170) | -0.368^**^ (0.161) | -0.523^**^ (0.165) | -0.240 (0.164) | -0.432^**^ (0.177) |
|  |  |  |  |  |  |  |  |  |  |
| Age | 0.005 (0.005) | 0.002 (0.005) | 0.016^**^ (0.005) | 0.017^**^ (0.005) | 0.021^**^ (0.005) | 0.023^**^ (0.006) | 0.018^**^ (0.005) | 0.012^**^ (0.005) | 0.017^**^ (0.006) |
|  |  |  |  |  |  |  |  |  |  |
| Income situation | 0.103 (0.070) | 0.226^**^ (0.082) | 0.116 (0.080) | 0.121 (0.077) | 0.206^**^ (0.082) | 0.006 (0.083) | 0.084 (0.086) | 0.096 (0.080) | 0.223^**^ (0.081) |
|  |  |  |  |  |  |  |  |  |  |
| Constant | -1.365 (0.832) | -1.046 (0.969) | -0.552 (0.967) | -1.828^**^ (0.772) | -3.189^**^ (0.847) | -1.498^*^ (0.861) | 0.480 (0.813) | -1.696^*^ (0.869) | -1.513 (0.939) |
| Pseudo *R*^2^ | 0.026 | 0.038 | 0.042 | 0.047 | 0.065 | 0.047 | 0.030 | 0.054 | 0.085 |
| Observations | 1003 | 989 | 990 | 1007 | 1022 | 983 | 993 | 981 | 977 |

*Note*: Logistic modeling coefficients with robust standard errors in parentheses. Reference category for education is “primary & lower secondary”. ^*^ *p* < 0.10, ^**^ *p* < 0.05

**AT14b**: Big Five of Personality and Covid-19-Intolerance (“Teacher”) in Switzerland, Spain and the United Kingdom

|  | **Switzerland** | | | **Spain** | | | **United Kingdom** | | |
| --- | --- | --- | --- | --- | --- | --- | --- | --- | --- |
| Pandemic phase | Spring 2020 | Winter 2020/2021 | Spring 2021 | Spring 2020 | Winter 2020/2021 | Spring 2021 | Spring 2020 | Winter 2020/2021 | Spring 2021 |
|  |  |  |  |  |  |  |  |  |  |
| Openness to experience | -0.792^*^ (0.443) | 0.175 (0.344) | 0.058 (0.368) | 0.535^*^ (0.300) | 1.042^**^ (0.328) | 0.873^**^ (0.322) | 0.678 (0.447) | 0.074 (0.300) | -0.051 (0.305) |
|  |  |  |  |  |  |  |  |  |  |
| Extraversion | -0.329 (0.264) | -0.372 (0.251) | -0.423 (0.278) | -0.423^*^ (0.250) | -0.709^**^ (0.322) | -0.119 (0.283) | -1.177^**^ (0.309) | -0.215 (0.209) | -0.130 (0.207) |
|  |  |  |  |  |  |  |  |  |  |
| Conscientiousness | 0.829^*^ (0.426) | 0.836^**^ (0.388) | 0.819^**^ (0.366) | 0.212 (0.336) | 1.825^**^ (0.376) | 1.690^**^ (0.390) | 0.755^*^ (0.440) | 0.479 (0.310) | 0.259 (0.314) |
|  |  |  |  |  |  |  |  |  |  |
| Agreeableness | 0.476 (0.387) | 0.824^**^ (0.384) | 1.665^**^ (0.414) | 0.885^**^ (0.423) | 0.337 (0.459) | 0.327 (0.460) | -0.148 (0.447) | 0.706^**^ (0.324) | 0.106 (0.336) |
|  |  |  |  |  |  |  |  |  |  |
| Neuroticism | 0.552^**^ (0.242) | -0.254 (0.228) | 0.469^**^ (0.238) | 0.330 (0.233) | -0.208 (0.260) | -0.150 (0.255) | 0.064 (0.305) | 0.443^**^ (0.205) | 0.259 (0.196) |
|  |  |  |  |  |  |  |  |  |  |
| Education |  |  |  |  |  |  |  |  |  |
|  |  |  |  |  |  |  |  |  |  |
| Upper, post secondary | 0.827^**^ (0.257) | 0.198 (0.242) | 0.613^**^ (0.285) | 0.080 (0.188) | -0.012 (0.201) | -0.241 (0.205) | 0.098 (0.195) | 0.398^*^ (0.207) | 0.243 (0.209) |
|  |  |  |  |  |  |  |  |  |  |
| Tertiary | 0.705^**^ (0.269) | 0.306 (0.244) | 0.651^**^ (0.288) | 0.246 (0.174) | 0.543^**^ (0.209) | 0.446^**^ (0.212) | 0.566^**^ (0.168) | 0.559^**^ (0.198) | 0.481^**^ (0.187) |
|  |  |  |  |  |  |  |  |  |  |
| Male | -0.118 (0.169) | -0.426^**^ (0.157) | 0.052 (0.159) | -0.021 (0.146) | 0.145 (0.161) | 0.181 (0.166) | -0.162 (0.149) | -0.117 (0.156) | 0.034 (0.155) |
|  |  |  |  |  |  |  |  |  |  |
| Age | 0.015^**^ (0.005) | 0.007 (0.004) | 0.018^**^ (0.005) | 0.014^**^ (0.005) | 0.010^*^ (0.006) | 0.022^**^ (0.006) | 0.020^**^ (0.005) | 0.031^**^ (0.006) | 0.018^**^ (0.005) |
|  |  |  |  |  |  |  |  |  |  |
| Income situation | 0.141^*^ (0.077) | 0.204^**^ (0.071) | 0.190^**^ (0.075) | 0.201^**^ (0.072) | 0.010 (0.074) | 0.014 (0.081) | 0.048 (0.067) | 0.056 (0.067) | 0.107 (0.069) |
|  |  |  |  |  |  |  |  |  |  |
| Constant | -1.298 (0.893) | -1.516^*^ (0.817) | -4.218^**^ (0.938) | -2.157^**^ (0.896) | -2.556^**^ (0.966) | -3.311^**^ (0.971) | -0.994 (1.046) | -2.719^**^ (0.799) | -1.040 (0.772) |
| Pseudo *R*^2^ | 0.038 | 0.053 | 0.062 | 0.030 | 0.067 | 0.070 | 0.044 | 0.043 | 0.022 |
| Observations | 990 | 1120 | 990 | 992 | 1002 | 1016 | 1000 | 1029 | 986 |

*Note*: Logistic modeling coefficients with robust standard errors in parentheses. Reference category for education is “primary & lower secondary”. ^*^ *p* < 0.10, ^**^ *p* < 0.05
